# Design and Development of Transient Sensing Devices for Healthcare Applications

**DOI:** 10.1002/advs.202307232

**Published:** 2024-03-14

**Authors:** Željko Janićijević, Tao Huang, Diana Isabel Sandoval Bojórquez, Taufhik Hossain Tonmoy, Salvador Pané, Denys Makarov, Larysa Baraban

**Affiliations:** ^1^ Institute of Radiopharmaceutical Cancer Research Helmholtz‐Zentrum Dresden‐Rossendorf e. V. 01328 Dresden Germany; ^2^ Multi‐Scale Robotics Lab (MSRL) Institute of Robotics & Intelligent Systems (IRIS) ETH Zürich Zürich 8092 Switzerland; ^3^ Institute of Ion Beam Physics and Materials Research Helmholtz‐Zentrum Dresden‐Rossendorf e. V. 01328 Dresden Germany

**Keywords:** biodegradable electronics, biosensors, biomedical devices, clinical diagnostics, healthcare applications, point‐of‐care diagnostics, wearable electronics

## Abstract

With the ever‐growing requirements in the healthcare sector aimed at personalized diagnostics and treatment, continuous and real‐time monitoring of relevant parameters is gaining significant traction. In many applications, health status monitoring may be carried out by dedicated wearable or implantable sensing devices only within a defined period and followed by sensor removal without additional risks for the patient. At the same time, disposal of the increasing number of conventional portable electronic devices with short life cycles raises serious environmental concerns due to the dangerous accumulation of electronic and chemical waste. An attractive solution to address these complex and contradictory demands is offered by biodegradable sensing devices. Such devices may be able to perform required tests within a programmed period and then disappear by safe resorption in the body or harmless degradation in the environment. This work critically assesses the design and development concepts related to biodegradable and bioresorbable sensors for healthcare applications. Different aspects are comprehensively addressed, from fundamental material properties and sensing principles to application‐tailored designs, fabrication techniques, and device implementations. The emerging approaches spanning the last 5 years are emphasized and a broad insight into the most important challenges and future perspectives of biodegradable sensors in healthcare are provided.

## Graphical abstract

This work offers a comprehensive and balanced overview of essential aspects in the design and development of transient sensing devices for healthcare applications. It critically evaluates recent advances, progressing from material properties and sensing principles to fabrication methods and application‐tailored sensor designs, with the intention of establishing pertinent guidelines for researchers and engineers.

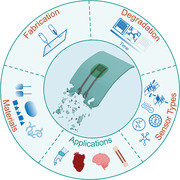

## 1 Introduction

As we approach the new era of advanced technologies in healthcare, highly accurate, personalized, and real‐time monitoring of health parameters becomes increasingly important in diagnostic, as well as in therapeutic applications.^[^ ^1^, ^2^, ^3^ ^]^ Therefore, there is an increasing demand for a new generation of wearable and implantable medical devices, contributing to the efficient prevention and management of diseases. An important requirement for such sensors is the seamless integration with the human body's tissues and organs, without altering their function. Such a level of integration is necessary to provide high quality and reliability of recorded data enabling prediction of patient outcomes and informed decision‐making. Many medical sensors are only required to operate effectively during a predetermined period defined by specific medical needs. After this period, removal of the device is desirable and may involve a procedure posing a significant health risk for the patient (such as surgical retrieval of sensors implanted at critical sites in the body). Prominent examples illustrating the requirement of temporary sensor operation include brain disease diagnostics,^[^ ^4^ ^]^ intracranial monitoring of recovery after traumatic brain injury,^[^ ^5^ ^]^ monitoring of blood flow after surgical interventions,^[^ ^6^ ^]^ and monitoring of healing in damaged tissues,^[^ ^7^, ^8^ ^]^ As the reliance on temporary electronic devices comprising personalized sensing systems for health, sports, and wellness purposes grows rapidly in modern society (including the healthcare sector), the produced waste originating from disposed electronics (e‐waste) is accumulating at an unprecedented pace and may become a significant hazard to humans and the environment.^[^ ^9^, ^10^ ^]^ Although low‐cost recycling and repurposing strategies have been demonstrated for some materials within temporary electronic devices,^[^ ^11^ ^]^ their large‐scale effectiveness remains limited.

The development of transient electronics and sensing devices relying on the use of biodegradable or bioresorbable materials^[^ ^10^, ^12^ ^]^ that can be naturally eliminated from the body or the environment, without the release of harmful by‐products, is one of the most attractive solutions to the aforementioned problems. Progress achieved through decades of researching degradable biomaterials, mostly developed for drug delivery^[^ ^13^ ^]^ or tissue regeneration^[^ ^14^ ^]^ applications, has enabled the emergence of biodegradable sensors and transient electronics suitable for healthcare monitoring applications. As a consequence of these development efforts, the global market for biodegradable electronic components and devices is projected to grow at a compound annual growth rate of 12.23% to reach $ 1.08 billion by 2027.^[^ ^15^ ^]^ The major driving forces for such a market will be the emerging applications aiming at reducing electronic waste, eliminating secondary surgery for medical implant retrieval, and protecting sensitive data.^[^ ^16^ ^]^ To address the market needs in the near future, it is desirable to realize wearable and implantable sensors in healthcare that are fully degradable while ensuring that the final degradation products do not cause harm to the patient and cumulatively introduce negative effects on the environment.

Generally, to construct a complete sensing device, it is necessary to integrate several crucial building blocks: a) the sensing module, b) the power source, c) the readout system, and d) the appropriate enclosure. The complexity of the device will depend on the sensing principles, location of use, and specific application demands. Transferring the design and development of the sensors and implants into the realm of biodegradable technology requires balanced trade‐offs between device miniaturization, suitable sensing performance, sufficient power supply for operation, and appropriate materials selection from a limited pool of candidates. The reliable functioning of biodegradable sensors requires a smart balance between mechanical, electrical, and optical performance including adequate matching of properties with the surrounding tissues and a controlled degradation process.

Considering that the first report on a partially resorbable transistor appeared in 2009,^[^ ^17^ ^]^ it is remarkable that (in less than 15 years) the development of advanced materials and improved fabrication techniques have enabled the production of almost all components necessary for fully biodegradable sensors. While individual components are available, assembling them into a fully functional sensing device remains challenging. For the complete realization of a new generation of fully biodegradable sensing devices, major hurdles to overcome include the development of soft and biodegradable highly conductive materials,^[^ ^18^ ^]^ effective miniaturization of electrical or optical power sources,^[^ ^19^, ^20^, ^21^ ^]^ efficient wireless communication^[^ ^22^ ^]^ and power transfer,^[^ ^23^ ^]^ and precise control (or programming) of degradation processes.^[^ ^24^, ^25^ ^]^

**Figure 1:**
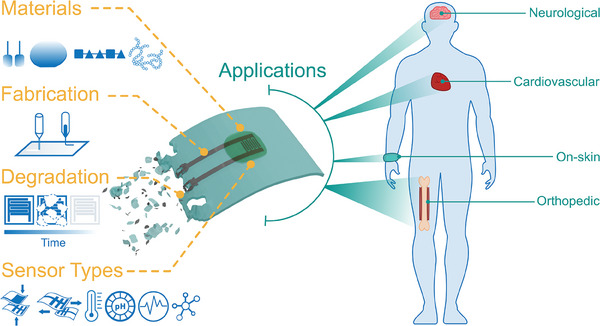
Conceptual overview encompassing the key topics to be considered for the design and development of transient sensing devices for healthcare applications.

To date, a plethora of wearable and implantable physical sensors have been realized in a biodegradable format to monitor pressure, strain, temperature, humidity, and electrophysiological signals. However, only a limited number of (bio‐)chemical sensors have been developed to monitor gases, pH, and relevant biomolecules. These physical and chemical sensors were intended for different areas of healthcare monitoring involving on‐skin, orthopedic, cardiovascular, and neurological applications (**Figure** **1**).

On the other hand, numerous impactful reviews were published on biodegradable and bioresorbable materials^[^ ^26^, ^27^, ^28^ ^]^ and biodegradable sensors^[^ ^29^, ^30^ ^]^ and implants,^[^ ^31^ ^]^ typically focusing on material properties and highlighting specific applications. In contrast, our mission here is to make an integrative ‘bottom‐up’ overview of the broader scope of the materials, fabrication techniques, and sensing principles to highlight and unveil their impact on the novel transient monitoring devices in the healthcare sector. Hence, here we stimulate the development of design rules for transient sensing systems, starting from the material level, followed by the key assembly and production concepts, and progress to the level of sensing devices oriented toward different applications (Figure 1). Therefore, this review offers complementary insights and a systematic balanced overview with tutorial elements, relevant for a broader audience of researchers and engineers interested in constructing biodegradable systems for healthcare monitoring. Our focus is on the critical assessment of recent advances and emerging approaches spanning the last 5 years while highlighting key development concepts and identifying future challenges and perspectives.

## 2 Materials for Biodegradable Sensors

The selection of materials for biodegradable sensors is limited by the specific set of desired properties required for suitable device performance. These materials should simultaneously satisfy the demands for safe degradation as well as for reliable performance during the designated measurement period. Desired material properties are often contradictory, and an adequate trade‐off should be considered from the early sensor design stages and later adapted or verified for the specific application.

**Scheme 1:**
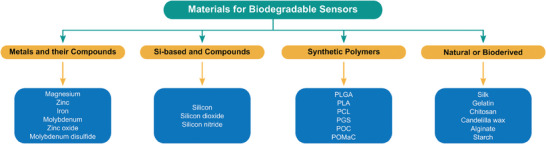
Overview of materials used in biodegradable sensors (classification and prominent examples).

Limited possibilities for selecting suitable fully biodegradable and bioresorbable materials have resulted in the use of various material types. An overview of the described material types with their classification and prominent examples is provided in **Scheme** **1**. These include inorganic materials, such as metals, some semiconductors, and their selected oxides, sulfides, and nitrides.^[^ ^33^, ^34^ ^]^ Additionally, organic synthetic materials, mainly embracing different classes of polymers, have been employed.^[^ ^18^, ^35^ ^]^ Furthermore, natural materials or their derivatives, such as certain amino acids, proteins, carbohydrates, and waxes have also been considered.^[^ ^36^, ^37^, ^38^, ^39^, ^40^, ^41^ ^]^ The selection of these materials requires careful consideration of the desired material properties required for optimal sensing performance, while simultaneously adhering to the limitations imposed by safe degradation. These factors are all defined by the specific application. The selection of materials is a critical step that will further influence sensor design, operational lifetime, and fabrication compatibility constraints.

Biocompatibility is the primary consideration during material selection for the development of biodegradable sensors.^[^ ^42^ ^]^ This feature must be maintained throughout their life cycle (including the safety of degradation products) while ensuring the structural and functional integrity of the device during its operational lifetime. It is vital to match the mechanical properties of biodegradable sensors with the surrounding tissues to avoid mechanical stress and tissue damage. Additionally, the dissolution properties and susceptibility to water penetration of the material need to be carefully considered. Depending on the application, a thorough assessment of the electrical, optical, and chemical properties of the material is crucial for optimizing sensor design.^[^ ^4^, ^6^, ^32^, ^39^ ^]^ Note that certain wearable biodegradable sensing devices, such as those designed for non‐invasive use on the skin, must satisfy significantly less stringent requirements compared to implantable devices that are designed to be resorbed within the body.

Degradation of the materials is typically first studied in vitro, where the application conditions (pressure, temperature, and ionic composition) are simulated.^[^ ^43^ ^]^ For instance, in the case of extended degradation processes, accelerated degradation at elevated temperatures can be used to estimate the degradation period and kinetics using Arrhenius scaling.^[^ ^21^, ^44^ ^]^ Subsequently, this investigation can be followed by in vivo studies of degradation using animal models, where different degradation dynamics are expected, and degradation products are eliminated due to the circulation of body fluids and metabolic processes.^[^ ^44^ ^]^ The biodegradation of wearable biodegradable sensors should occur naturally in the environment with the assistance of microorganisms and enzymes.^[^ ^45^ ^]^

In the following sections, we comprehensively review the main material classes used in biodegradable sensors for healthcare monitoring. Emphasis is placed on their chemical properties, potential applications in sensor components, and degradation behavior.

### 2.1 Metals and their Compounds

Metals typically play an indispensable role in the configuration of some electronic components such as antennae, resistors, and capacitors, offering the base for electrodes, contacts, and interconnects. Metallic materials are commonly applied in the form of thin films or foils processed using traditional micro‐ and nano‐fabrication techniques. Recently, there has been a growing significance of powder and ink formulations that are blended with polymeric matrices and binders. These alternatives are compatible with cost‐effective printing techniques.^[^ ^37^, ^46^, ^47^, ^48^, ^49^ ^]^

There is a limited number of degradable metals with biocompatible properties and safe degradation products, making them suitable for healthcare applications. This group of metals includes magnesium (Mg), zinc (Zn), molybdenum (Mo), tungsten (W), and iron (Fe).^[^ ^33^ ^]^ In aqueous media, these metals dissolve via hydrolysis into ions, oxides, and hydroxides or acids, depending on the chemical environment.^[^ ^25^ ^]^ **Table** **1** summarizes the key electrical and degradation properties of biodegradable metals. Degradation proceeds through electrochemical corrosion and mechanical disruption of the structure caused by cracking and the formation of gaseous products (e.g., hydrogen gas, H_2_). The process of dissolution can be partially engineered by tuning microstructural properties (particle or grain size, texture, defects, and porosity). Corrosion processes occurring simultaneously for the defined galvanic pair of metals can be even exploited for sensing the amount of dissolved gaseous species (such as oxygen, O_2_).^[^ ^50^ ^]^ In specific cases, where big amounts of metal are required (electrode arrays with large area or thick metallic regions for mechanical support), alloying may be preferred to achieve desired and controllable degradation rates^[^ ^51^, ^52^, ^53^ ^]^ together with improved mechanical properties.^[^ ^54^ ^]^

**Table 1:** Electrical conductivity and degradation properties of common biodegradable metals.

Metal	Bulk Conductivity [MS/m]^[^ ^55^ ^]^	Degradation Chemistry^[^ ^56^ ^]^	Degradation Medium	Degradation Rate [nm per day]^[^ ^56^, ^57^ ^]^
Mg	23	Mg + 2H_2_O → Mg(OH)_2_ + H_2_	PBS (pH 7.4, 37 °C)	≈1700 (deposited layer)
Zn	17	Zn + 2H_2_O → Zn(OH)_2_ + H_2_	PBS (pH 7.4, 37 °C)	≈3500 (thin foil)
Fe	10	Fe + 1/2O_2_ + H_2_O → Fe(OH)_2_	PBS (pH 7.4, 37 °C)	≈80 (thin foil)
Mo	20	Mo + 3H_2_O → MoO_3_ + 3H_2_, MoO_3_ + 2OH^−^ → HMoO_4_^−^ + H_2_O + e^−^	PBS (pH 7.4, 37 °C)	≈20 (thin foil)
W	20	W + 3H_2_O → WO_3_ + 3H_2_, WO_3_ + H_2_O → H_2_WO_4_	PBS (pH 7.4, 37 °C)	≈150 (thin foil)

There are significant differences between metal candidates for the construction of biodegradable sensors in terms of conductivity and degradation properties (see Table 1), with each one offering specific advantages. Selection of the appropriate degradable metal depends on the device function, operational lifetime, application site, and physiological tolerance to the uptake of degradation products. We note that Table 1 contains one elemental ferromagnetic material (Fe) also forming biocompatible and biodegradable oxides. This opens an additional opportunity to realize biodegradable magnetoresistive magnetic field sensing devices.

Among biodegradable metals, Mg has been the most abundantly used due to its compatibility with conventional micro‐ and nano‐processing methods, excellent electrical conductivity, high energy density, and rapid degradation, making it particularly suitable for sensing elements and device components where it is not directly exposed to biofluids.^[^ ^6^, ^7^, ^19^ ^]^ Mg was employed in a variety of physical sensors (e.g., pressure,^[^ ^7^, ^36^, ^58^, ^59^ ^]^ strain,^[^ ^7^ ^]^ and temperature^[^ ^60^, ^61^, ^62^ ^]^) where it was used as a wire,^[^ ^6^, ^59^ ^]^ electrode,^[^ ^7^, ^39^, ^59^, ^60^, ^61^, ^63^ ^]^ coil,^[^ ^6^, ^59^, ^60^, ^61^ ^]^ substrate,^[^ ^64^ ^]^ and sensing element,^[^ ^62^ ^]^ but also in power sources such as batteries, where it served as anode layer.^[^ ^19^, ^65^ ^]^ For example, a patterned evaporated Mg layer was used to form electrodes for capacitive sensing of pressure and strain, enabling real‐time monitoring of tendon healing.^[^ ^7^ ^]^ Mg foil was used to pattern a coil inductor and electrodes for LC‐resonance‐based bioresorbable body temperature sensors.^[^ ^60^ ^]^ Kirigami‐patterned Mg foil was also used to create a stretchable anode in a highly deformable and biodegradable battery.^[^ ^65^ ^]^ While rapid degradation of Mg is convenient for tuning the operational lifetime of biodegradable sensors, significant accumulation of Mg degradation products (OH^−^ and H_2_) can induce toxicity^[^ ^43^, ^66^ ^]^ and the amount of released Mg should be carefully considered.

The properties of Zn are similar to those of Mg in terms of degradation behavior. However, Zn exhibits lower bulk conductivity (see Table 1). Zn has been mainly used to construct electrodes,^[^ ^2^, ^59^, ^67^ ^]^ wires,^[^ ^62^ ^]^ and interconnects^[^ ^67^ ^]^ in physical sensors. In addition, it has been also employed as a temperature sensor,^[^ ^41^ ^]^ and as an electrode for electrophysiological monitoring.^[^ ^41^ ^]^ Interestingly, microparticles and nanoparticles of Zn are emerging as attractive fillers in paste and ink formulations, which are compatible with different printing approaches used to fabricate biodegradable conductors.^[^ ^68^, ^69^, ^70^, ^71^ ^]^

In contrast to Mg and Zn, Fe has a slower (see Table 1) and less predictable degradation rate,^[^ ^56^, ^72^ ^]^ mainly because of the low solubility of produced iron oxide species in water. Consequently, Fe is not as common in emerging biodegradable sensors as Mg and Zn, although iron‐based particles have been employed as catalysts in implantable biosensors^[^ ^73^ ^]^ and used in magnetic field concentrators.^[^ ^23^ ^]^ Interestingly, thin crystalline films of elemental Fe also show great potential for constructing biodegradable magnetoresistive sensors.^[^ ^74^, ^75^ ^]^ In combination with Zn, Fe can form bilayer electrodes suitable for degradable pressure sensors.^[^ ^76^ ^]^

Generally, Mg and Zn offer fast degradation rates compared to Fe but also suffer from inhomogeneous dissolution, which somewhat limits their use in sensors. The dissolution of Mg, Zn, and Fe is strongly influenced by the adsorption of biomolecules (such as amino acids, proteins, and lipids) and the presence of chloride ions (Cl^−^) in the physiological environment, affecting the stability of their oxide and hydroxide surface layers.^[^ ^77^ ^]^ The adsorption of Cl^−^ can cause the degradation of the hydroxide which often acts as a protective layer, leading to pitting corrosion and fragmentation of the degradation products.^[^ ^77^ ^]^ Such behavior is particularly common for Mg.^[^ ^78^ ^]^ Zn and Fe dissolve in a non‐uniform manner through competing degradation reactions leading to the formation of oxides and hydroxides with different rates of dissolution in the physiological environment.^[^ ^25^ ^]^ Degradation of Zn forms a mixed layer of ZnO and Zn(OH)_2_ with porous morphology and non‐uniform chemical composition, while Fe can degrade by forming a multitude of oxides (FeO, Fe_2_O_3_, and Fe_3_O_4_) and hydroxides (Fe(OH), Fe(OH)_2_, and Fe(OH)_3_).^[^ ^56^, ^72^ ^]^

As an alternative, components made from transition metals such as Mo and W can survive longer in physiological environments showing gradual degradation, when directly exposed to biofluids. Degradation of Mo and W is dependent on oxygen dissolution in the surrounding medium as well as on the degradation rates of their oxide intermediates. Although Mo and W degrade by initially forming a mixture of oxides under physiological conditions, resulting oxides exhibit similar rates of dissolution allowing for steady and consistent removal of thin corroded layers.^[^ ^25^, ^79^ ^]^

Mo electrode patterns enabled electrocorticogram recording in rats while maintaining stable impedance for more than 3 days when exposed to PBS^[^ ^4^ ^]^ as well as chronic wound healing assessment in mice during a few weeks by monitoring impedance changes.^[^ ^8^ ^]^ Thin W film in a transient light‐emitting diode showed predictable and gradual degradation for more than one week in contact with PBS.^[^ ^21^ ^]^ Even as electrodes in microsupercapacitors, Mo and W showed good electrochemical performance and survived for several days in contact with PBS or hydrogel electrolyte.^[^ ^80^ ^]^ Therefore, these metals have the potential to make an impact in implantable bioresorbable sensors. Mo served as a wire,^[^ ^81^ ^]^ contact layer,^[^ ^20^ ^]^ electrode for electrophysiological and pressure signal recording,^[^ ^4^ ^]^ and electrode material for power sources (degradable batteries^[^ ^19^, ^65^ ^]^ and supercapacitors^[^ ^80^ ^]^) or dissolvable gas sensors.^[^ ^50^ ^]^ W is not used as commonly as Mo and it typically performs as a contact layer component in the form of thin films^[^ ^21^ ^]^ or as a filler in conductive bioresorbable inks^[^ ^82^ ^]^ and composites.^[^ ^37^, ^83^ ^]^ However, it was also utilized in some degradable power supplies as an electrode material.^[^ ^80^ ^]^

Notably, Mg, Zn, Fe, and Mo represent essential metals for human metabolism.^[^ ^84^, ^85^, ^86^, ^87^ ^]^ Namely, Mg is an important constituent of bone tissue.^[^ ^78^ ^]^ It also plays a significant role in many cellular processes, such as the synthesis of biomolecules (proteins and nucleic acids) and cell membrane stabilization.^[^ ^84^ ^]^ Zn is an indispensable element for the regulation of physiological processes in the human body, such as enzymatic reactions, gene expression, immune response, and antioxidant defense, which can act as a structural component, catalytic factor, or signaling mediator in intra‐ and inter‐cellular communication.^[^ ^85^, ^88^ ^]^ Fe is required for the synthesis of hemoglobin, redox reactions, and proliferation of cells.^[^ ^87^ ^]^ Finally, Mo is a key constituent of enzymes catalyzing various reactions necessary for the regulation of carbon, sulfur, and nitrogen metabolism.^[^ ^86^, ^89^ ^]^ Importantly, Fe metabolism is a highly regulated semi‐closed system operating without the mechanism of active excretion from the body.^[^ ^87^ ^]^ Conversely, the excess of Mg, Zn, and Mo can be successfully excreted from the body via urine and feces.^[^ ^90^, ^91^, ^92^, ^93^ ^]^

Some metal compounds such as molybdenum trioxide (MoO_3_), molybdenum disulfide (MoS_2_), and zinc oxide (ZnO), emerged as materials of great interest for biodegradable sensor construction. These materials can be found in various sensor components.^[^ ^19^, ^21^, ^34^, ^65^, ^94^, ^95^ ^]^ For example, MoS_2_ is an interesting Mo‐based compound with especially favorable properties when prepared in the form of thin films as 2D material, such as transparency, flexibility, and extraordinary mechanical strength.^[^ ^96^ ^]^ The comprehensive study of Chen et al.^[^ ^34^ ^]^ shows the semiconductive behavior of MoS_2_ monolayers, which gradually and safely biodegrade via hydrolysis under physiological conditions in vitro (after more than 2 months) and in vivo (after 4 weeks). Notably, the degradation rate could be modulated by adjusting the grain size and defect density in the films. In the same study, the authors demonstrated the utility of fully resorbable MoS_2_ layers by constructing sensors capable of measuring pressure, temperature, strain, and acceleration.

MoO_3_ is also an attractive cathode material for biodegradable batteries due to its high‐energy density and layered structure accessible for the intercalation of foreign ions.^[^ ^65^ ^]^ When coupled to a Mg anode, the MoO_3_ cathode forms a Mg‐MoO_3_ cell that can operate at a theoretical voltage similar to commercial alkaline batteries (1.6 V).^[^ ^19^, ^65^ ^]^ Furthermore, MoO_3_ is readily soluble in water (≈1 g L^−1^) and exhibits good biocompatibility, when its concentration is well‐controlled.^[^ ^19^, ^97^ ^]^ The properties of MoO_3_ cathode in biodegradable batteries can be enhanced by forming a paste or composite with hydrolytically degradable polymers serving to increase the effective surface area and slow down the dissolution of MoO_3_.

Finally, ZnO is a well‐known semiconductor material with a wide direct bandgap (3.37 eV) and large exciton‐binding energy (60 meV) interesting for the fabrication of electronic and optical devices.^[^ ^98^ ^]^ ZnO is relatively easy to fabricate and process using a variety of techniques. It also exhibits high electron mobility, excellent luminous transmittance, low dielectric constant, piezoelectric properties, low toxicity, and biocompatibility.^[^ ^94^, ^99^ ^]^ Because of their promising properties, ZnO‐based materials in various formats were explored as components in diverse biochemical sensors,^[^ ^94^ ^]^ bioresorbable transistors and piezoelectric energy harvesters,^[^ ^95^ ^]^ and transient bioresorbable LEDs.^[^ ^21^ ^]^ With a rate of ≈ 100 nm per day, ZnO films gradually dissolve under physiological conditions in vitro (PBS, pH 7.4, 37 °C) into soluble Zn^2+^ ions or moderately soluble hydroxides depending on the amount of ZnO, volume of the medium, and reaction conditions.^[^ ^21^, ^95^ ^]^

Metals and their selected compounds described in this section are important constituents of biodegradable sensing devices, mostly because of their outstanding electrical properties, which cannot be replaced yet by other material classes. The use of these materials is typically limited by electrochemical corrosion and mechanical fragmentation, often occurring rapidly after exposure to physiologically relevant fluids. Therefore, they are more abundant in physical sensors where encapsulation is easy to achieve and direct exposure to the chemically active environment is not necessary. Different strategies are actively explored to control the degradation rate and effectively exploit metal‐based materials in electrophysiological and electrochemical sensors.

### 2.2 Silicon‐based Materials and Compounds

Because of the limited availability and importance of semiconducting materials in biodegradable sensing devices, thin monocrystalline or porous Si‐based materials may also be implemented in biodegradable sensors. For instance, the dissolution of thin monocrystalline Si layers in biologically relevant fluids during timescales suitable for sensor degradation was a significant milestone.^[^ ^100^ ^]^ The use of thin films or membranes (in the range from ≈50 nm to ≈5 µm) made from Si and its compounds rather than bulk Si, is important for the reduction of the degradation time of Si‐based sensor components as well as for the production of mechanically softer and more flexible layers compatible with biological tissues. Detailed knowledge about the precise processing of Si‐based materials^[^ ^101^ ^]^ coming from commercial electronics, additionally unlocks the potential for constructing high‐performance biodegradable sensing devices using foundry‐compatible techniques^[^ ^101^ ^]^ and multilayer structuring to integrate heterogenous transient materials.^[^ ^102^ ^]^ In the following, we review the properties and uses of Si‐based materials as device components in the context of biodegradable sensors. Key functional and degradation properties of Si‐based materials are provided in **Table** **2**.

**Table 2:** Functional and degradation properties of common biodegradable Si‐based materials.

Material	Functional Properties	Degradation Chemistry	Degradation Medium	Degradation Rate [nm per day]
Si	Compatible with micro‐/nano‐fabrication; Piezoresistive; Rigid as a bulk material; Slow degradation	Si + 4H_2_O → Si(OH)_4_ + H_2_^[^ ^33^ ^]^	PBS (pH 7.4, 37 °C)	≈4.5^[^ ^100^ ^]^
p‐Si	Simple to fabricate; High surface area; Rapid degradation; Easily tunable chemical and optical properties	Si + 4H_2_O → Si(OH)_4_ + H_2_^[^ ^33^ ^]^	Artificial cerebrospinal fluid (37 °C)	≈9000^[^ ^81^ ^]^
SiO_2_	Compatible with micro‐/nano‐fabrication; Tunable (slow) degradation; Thermal stability	SiO_2_ + 2H_2_O → S(OH)_4_^[^ ^103^ ^]^	PBS (pH 7.4, 37 °C)	0.003‐^[^ ^104^ ^]^
Si_3_N_4_	Compatible with micro‐/nano‐fabrication; Slow degradation; Thermal stability	1) Si_3_N_4_ + 6H_2_O → 3SiO_2_ + 4NH_3_ 2) SiO_2_ + 2H_2_O → S(OH)_4_^[^ ^33^ ^]^	PBS (pH 7.4, 37 °C)	0.16/0.85^[^ ^104^ ^]^

Si can be a constituent of biodegradable sensors in different formats including monocrystalline micromembranes (MM) (thickness: ≈µm) and nanomembranes (NMs) (approximate thickness range: 50‐ nm), and porous Si (p‐Si). These formats have found diverse applications in biodegradable sensing devices. Si MM was used as a protective impermeable water barrier in intracranial pressure sensors,^[^ ^64^ ^]^ while microlayers of Si were also employed as supports to construct Fabry‐Perot cavities for the measurement of localized subcutaneous tissue temperatures^[^ ^105^ ^]^ and continuous dual monitoring of intracranial pressure and temperature.^[^ ^5^ ^]^ Si NMs have found suitable use in a variety of biodegradable sensors as pressure sensing elements for optical^[^ ^5^ ^]^ and electrical^[^ ^64^ ^]^ intracranial pressure monitoring, strain and temperature gauges for highly accurate intracranial pressure and temperature monitoring,^[^ ^106^ ^]^ bioresorbable photodetectors for spectroscopic determination of oxygenation, temperature, melanin, and Ca^2+^,^[^ ^2^ ^]^ optical filters for biodegradable light‐emitting diodes,^[^ ^21^ ^]^ sensing layer supports in chemical sensors for dopamine level measurement,^[^ ^73^ ^]^ and components of gas sensors for NO_x_ species detection.^[^ ^107^ ^]^ p‐Si with nanoscale porosity was used as a supporting substrate in an intracranial pressure sensor^[^ ^81^ ^]^ and as a starting material for the fabrication of an in vivo pH sensor.^[^ ^32^ ^]^

Si degradation in aqueous media under physiological conditions occurs via hydrolysis as the main mechanism (see Table 2) and proceeds until complete dissolution to final degradation products typically within days or weeks (for thin Si films).^[^ ^108^ ^]^ Hwang et al. quantified the degradation rate of monocrystalline Si nanomembranes (Si NMs) under physiological conditions in vitro as 4.5 nm per day.^[^ ^100^ ^]^ The degradation rate can be tuned to decelerate by introducing dopants with different concentrations.^[^ ^109^ ^]^ The degradation rate can be also affected by the parameters of the surrounding medium (e.g., pH, temperature, and composition).^[^ ^25^ ^]^ Interestingly, degradation times increase due to protein adsorption and decrease due to the presence of Ca^2+^ ions.^[^ ^110^ ^]^ Nevertheless, Si NMs degrade controllably and uniformly through surface erosion.^[^ ^20^, ^33^ ^]^ Si layers with different crystal structures degrade at comparable rates following similar chemistry of the process (polycrystalline Si: 2.8 nm per day; amorphous Si: 4.1 nm per day).^[^ ^111^ ^]^

Silicon dioxide (SiO_2_) is well‐known in electronics for its dielectric properties, and because of its ability to achieve slow and uniform degradation, it became a very popular choice for encapsulation and dielectric layers in cases when polymer dielectrics are not sufficient to achieve adequate long‐term resistance to swelling and water penetration. As a reliable encapsulation material, it is well‐suited for adjustment of the device lifetime by tuning the passive transiency of the enclosure layer, which is a common approach.^[^ ^31^ ^]^ In this case, the degradation time of the enclosure is determined by the thickness of the fabricated SiO_2_ layer and the degradation rate (see Table 2) in the surrounding physiological medium. Different types of SiO_2_ have been used in diverse biodegradable sensors as encapsulation and protective layers,^[^ ^2^, ^62^, ^73^, ^106^ ^]^ interlayer dielectrics in dopamine and NO_x_ sensors,^[^ ^73^, ^107^ ^]^ support structures in intracranial pressure sensors,^[^ ^106^ ^]^ light confinement elements in bioresorbable spectroscopic chemical sensors,^[^ ^2^ ^]^ distributed Bragg reflector components in implantable tissue temperature sensors,^[^ ^105^ ^]^ and nanoporous scaffolds for in vivo pH sensors.^[^ ^32^ ^]^ The use of amorphous silica for adhesive layers is also important in some optical sensors.^[^ ^5^ ^]^

Dissolution of SiO_2_ follows the hydrolysis mechanism similar to Si,^[^ ^103^ ^]^ although it typically proceeds at much lower rates, depending on the layer formation method (e.g., thermal growth, plasma‐enhanced chemical vapor deposition, or electron‐beam evaporation) and the resulting properties of the obtained material such as density and stoichiometry.^[^ ^104^ ^]^ Obtained SiO_2_ layers with the highest density and most favorable stoichiometry exhibit the slowest degradation rate under physiological conditions (dry thermal growth, Si: O = 1: 2, 0.003 nm per day), and the opposite holds for oxygen‐rich SiO_2_ layers with considerably lower density (electron‐beam evaporation, Si: O = 1: 2.2, ≈10 nm per day).^[^ ^104^ ^]^

Another interesting compound is silicon nitride (Si_3_N_4_) used in a similar manner and often in combination with SiO_2._ It was used as an encapsulation layer in pressure^[^ ^59^ ^]^ and temperature^[^ ^62^ ^]^ sensors, as well as a distributed Bragg reflector component layer alternated with SiO_2_ in implantable tissue temperature sensors.^[^ ^105^ ^]^

Si_3_N_4_ layers degrade via hydrolysis in a two‐step process (see Table 2). Degradation rates reported for Si_3_N_4_ (two types of chemical vapor deposition, 0.16 and 0.85 nm per day) depend on the method of layer formation and resulting material properties in a similar manner as for SiO_2_ layers.^[^ ^104^ ^]^

From the aforementioned applications in many physical and a few chemical biodegradable sensors, it is clear that Si‐based materials open the possibility of utilizing more sophisticated optical sensing principles in addition to the predominantly exploited electromechanical sensing approaches in metal‐based biodegradable sensors. As can be observed from their dissolution chemistry, the main degradation product of Si‐based materials is silicic acid. Importantly, an excess amount of silicic acid does not accumulate in the body, but it is rather excreted in urine.^[^ ^106^ ^]^ However, the design of bioresorbable implants should consider limiting the degradation rates to avoid harmful effects of gaseous side products of degradation (H_2_ and NH_3_). Because of the extensive knowledge about precise processing and degradation control, Si‐based materials remain indispensable components of advanced biodegradable sensing devices requiring high accuracy, intricate design, and precise geometry. Significant efforts have been also invested in developing Ge‐based material alternatives for biodegradable sensors with similar requirements in recent years.^[^ ^112^ ^]^ The main disadvantages of Si‐based materials are their slow degradation and lack of mechanical flexibility (except for thin films). These disadvantages are typically addressed by employing polymeric materials, which we review in the following sections.

### 2.3 Synthetic Polymers

Together with inorganic materials, synthetic polymers are the most abundant building blocks in biodegradable sensors, commonly acting as substrates, adhesives, dielectrics, and encapsulation layers. Their properties are modulated by molecular design and processing techniques to tune their degradation behavior and mechanical properties (e.g., flexibility and stretchability).^[^ ^18^, ^45^, ^113^ ^]^ In principle, the biodegradation of polymers can occur through hydrolytic, enzymatic, and oxidative routes^[^ ^114^ ^]^ offering different degradation rates. In aqueous media, the degradation chemistry of commonly used polymers relies on cleaving chemical linkages (such as ester, amide, anhydride, acetal, carbonate, urethane, imide, and imine) by hydrolysis.^[^ ^45^ ^]^ The properties of common synthetic and natural polymers relevant to the fabrication and application of biodegradable sensors are listed in **Table** **3**.

**Table 3:** Mechanical, thermal, and degradation properties of common biodegradable polymers.

Polymer	Tensile Modulus [GPa]	Thermal Properties [°C]	Degradation Medium	Degradation Time or Rate
PLA	1.2‐3.0^[^ ^139^ ^]^	T_g_ ^a^ ^)^: 54, T_m_ ^b)^: 170^[^ ^139^ ^]^	PBS, 74 °C	8 days (50% mass loss)^[^ ^140^ ^]^
PLGA (50:50)	0.04‐0.05^[^ ^62^ ^]^	T_g_: 40^[^ ^62^ ^]^	PBS, 37 °C	2‐4 weeks^[^ ^62^ ^]^
PCL	0.4^[^ ^139^ ^]^	T_g_: ‐62, T_m_: 57^[^ ^139^ ^]^	PBS, 37 °C	14 h^[^ ^62^ ^]^
POC	0.0028‐0.00644^[^ ^62^ ^]^	T_g_: −10^[^ ^62^ ^]^	PBS, 37 °C	25 weeks^[^ ^62^ ^]^
PGS	0.00007.00705^[^ ^141^ ^]^	T_g_: −32.2 to −25.1^[^ ^141^ ^]^	In vivo	2 weeks (70% mass loss)^[^ ^62^ ^]^
POMaC	0.00003.00154^[^ ^135^ ^]^	–	PBS, 37 °C	10 weeks (77% mass loss)^[^ ^135^ ^]^
PHB/PHV	0.0009^[^ ^6^ ^]^	T_g_: −5^[^ ^142^ ^]^	–	3 months^[^ ^53^ ^]^
PVA	0.0017‐0.025^[^ ^143^ ^]^	T_g_: 85, T_m_: 230^[^ ^144^ ^]^	In vivo	2 years^[^ ^145^ ^]^
PBAT	0.5^[^ ^62^ ^]^	T_g_: 53^[^ ^62^ ^]^	–	11 weeks^[^ ^62^ ^]^
Cellulose	135^[^ ^146^ ^]^	T_g_: 250^[^ ^147^ ^]^	Enzyme‐based medium	≈0.3 nm/min^[^ ^148^ ^]^
(Na‐)CMC	0.000001^[^ ^149^ ^]^	T_g_: 79^[^ ^149^ ^]^	Water	<10 min^[^ ^150^ ^]^
Silk	5^[^ ^144^ ^]^	T_g_: 217^[^ ^151^ ^]^	Protease solution	4 weeks^[^ ^152^ ^]^
Gelatin	0.0000393^[^ ^153^ ^]^	T_g_: −15^c)^ ^[^ ^154^ ^]^	Mineral medium	<10 days^[^ ^67^ ^]^
Chitosan	65^[^ ^155^ ^]^	T_g_: 140^[^ ^156^ ^]^	Enzyme‐based medium	20 days (80% mass loss)^[^ ^157^ ^]^
Candelilla wax	0.0001^[^ ^158^ ^]^	T_g_: 35.58^[^ ^158^ ^]^	In vivo	3 months (3.5% thickness loss)^[^ ^37^ ^]^

^a)^ T_g_: glass transition temperature;

^b)^ T_m_: melting temperature;

^c)^ Strongly dependent on water content

The most exploited synthetic polymers in biodegradable sensing devices are poly(esters) including poly(lactide) (PLA), poly(lactide‐*co*‐glycolide) (PLGA), poly(caprolactone) (PCL), and their different combinations or blends.^[^ ^4^, ^23^, ^32^, ^50^, ^60^, ^73^, ^76^, ^115^ ^]^ In addition to their common use in degradable sensors for healthcare applications, these polymers were widely studied as biodegradable materials for other biomedical applications, such as drug delivery and tissue regeneration,^[^ ^61^, ^116^, ^117^, ^118^ ^]^ and their safe degradation is well‐established.^[^ ^119^ ^]^ PLA is commonly used as a substrate, e.g., in dissolved oxygen and humidity sensors,^[^ ^50^, ^120^ ^]^ but it was also utilized as a component of specific encapsulation and nanostructured triboelectric layers in dynamic pressure sensors for postoperative monitoring of cardiovascular function,^[^ ^63^ ^]^ a spacer in temperature sensors for local body temperature measurements,^[^ ^60^ ^]^ and an optical nanowire in chemical sensing of pH and cytochrome c.^[^ ^115^ ^]^ PLA can be synthesized from two stereoisomers of lactic acid, d‐lactide, and l‐lactide with different proportions.^[^ ^121^ ^]^ Homopolymer poly(L‐lactide) (PLLA) degrades significantly slower than heteropolymer forms of PLA.^[^ ^122^ ^]^ PLLA has been employed as a substrate or one of its components in pressure sensors for intracranial and tendon monitoring^[^ ^4^, ^7^ ^]^ as well as an electrical insulation layer in wireless and biodegradable sensors for the monitoring of blood flow.^[^ ^6^ ^]^ In addition, PLLA can be engineered to exhibit interesting piezoelectric properties under specific conditions,^[^ ^123^, ^124^ ^]^ which can be exploited, e.g., for environmentally friendly sensors in force myography.^[^ ^125^ ^]^ PLGA is a popular substrate^[^ ^2^, ^32^, ^59^, ^60^, ^105^, ^107^ ^]^ and encapsulation material^[^ ^60^, ^76^ ^]^ used in electromechanical wearable and implantable pressure sensors, bioresorbable photonic devices for pressure, temperature, and chemical sensing, in vivo pH sensors, and gas sensors. It has been also employed as an adhesive interlayer in intracranial pressure sensors,^[^ ^59^, ^64^ ^]^ as an optical fiber in bioresorbable pressure, temperature, and spectroscopic chemical sensors,^[^ ^2^, ^5^ ^]^ and as a pressure‐sensitive layer in high‐performance wearable pressure sensors.^[^ ^126^, ^127^ ^]^ PLGA is synthesized by combining different proportions of lactide and glycolide components of different hydrophobicity, enabling both the control over hydrolysis rate and water penetration and, in turn, the programming of its degradation time.^[^ ^128^, ^129^ ^]^ PCL is a polymer with low glass transition and melting point temperatures, excellent blend‐forming ability, and a typically slower degradation rate compared to PLA.^[^ ^121^ ^]^ It was used as a substrate in sensors for transient dopamine monitoring^[^ ^73^ ^]^ and electrode arrays for signal recording in the brain when combined with PLLA,^[^ ^4^ ^]^ as well as the nanofibrous membrane in combination with PLGA for pressure sensors.^[^ ^76^ ^]^

Another important class of polymeric materials for biodegradable sensors are common biodegradable elastomers such as poly(1,8‐octanediol‐*co*‐citrate) (POC), poly(glycerol sebacate) (PGS), and poly[octamethylene maleate (anhydride) citrate] (POMaC). POC was exploited as an encapsulation material in biodegradable and stretchable pressure sensors^[^ ^130^ ^]^ and as an elastic adhesive layer in self‐powered dynamic pressure sensors for cardiovascular monitoring.^[^ ^63^ ^]^ PGS was first reported by Wang et al. as a cost‐effective, tough, and flexible biodegradable polymer.^[^ ^131^ ^]^ It typically degrades in vivo via a surface erosion mechanism, while retaining its shape and structural integrity,^[^ ^132^ ^]^ although further engineering of its mechanical and degradation properties is also possible.^[^ ^133^ ^]^ PGS was used for a stretchable non‐sticking layer allowing electrode sliding in biodegradable strain sensors for orthopedic applications,^[^ ^7^ ^]^ structured pressure‐sensitive dielectric layers of the capacitors in implantable sensors for pressure, strain, and blood flow monitoring,^[^ ^6^, ^7^ ^]^ supporting encapsulation or backbone material in stretchable biodegradable batteries,^[^ ^65^ ^]^ and electrospun flexible degradable substrates for temperature and strain sensing when mixed with PCL.^[^ ^134^ ^]^ POMaC is a soft stretchable polymer able to mimic the mechanical properties of many soft tissues, and its properties can be adjusted at the synthesis level through a dual cross‐linking mechanism and monomer composition.^[^ ^135^ ^]^ It degrades via a surface erosion mechanism similar to PGS.^[^ ^135^, ^136^ ^]^ POMaC was explored for soft packaging layers in biodegradable pressure and strain sensors for orthopedic and cardiovascular applications,^[^ ^6^, ^7^ ^]^ as well as a component of a strain sensor used to monitor tendon healing.^[^ ^7^ ^]^

Poly(hydroxybutyrate)/poly(hydroxyvalerate) (PHB/PHV) copolymers belong to a class of resorbable poly(esters) derived from microorganisms where they play a role in intracellular storage.^[^ ^121^ ^]^ These polymers are flexible and typically stiffer than PGS or POMaC when applied as thin films.^[^ ^6^ ^]^ PHB/PHV were employed as stiffer packaging materials reducing motion or pressure interference during the sensing of arterial pulsation^[^ ^6^ ^]^ or as components of triboelectric nanogenerators used for electrical stimulation of nerve cells.^[^ ^137^ ^]^ PHB composite with cellulose nanocrystals was also used for transient and wearable triboelectric nanogenerators, which are capable of self‐powered monitoring of human movement.^[^ ^138^ ^]^

Several other polymers play a significant role in biodegradable sensors, including poly(vinyl alcohol) (PVA), poly (ethylene glycol) (PEG), and poly(butylene adipate‐*co*‐terephthalate) (PBAT). PVA is a water‐soluble polymer^[^ ^159^ ^]^ commonly used as a cross‐linked hydrogel for long‐term implants in vivo, but some reports show that it can be fully degraded in vitro when applied in the form of thin films.^[^ ^160^ ^]^ It can be fully excreted and eliminated in vivo,^[^ ^161^ ^]^ as well as enzymatically degraded in an aqueous environment by bacterial strains secreting PVA oxidase, dehydrogenase, and hydrolase.^[^ ^162^ ^]^ PVA was used as a substrate^[^ ^76^ ^]^ or nanofibrous layer^[^ ^126^ ^]^ in high‐performance wearable and degradable pressure sensors, as well as a metal/polymer adhesive layer in highly sensitive and flexible pressure sensors for cardiovascular monitoring.^[^ ^163^ ^]^ It is also a constituent of ionically conductive hydrogels which are used to construct strain sensors for human motion monitoring with rapid response.^[^ ^164^ ^]^ PEG is a non‐toxic biodegradable material commonly used in the form of hydrogels, which is sensitive to oxidative degradation^[^ ^165^ ^]^ and its degradation products can be excreted in vivo through the kidneys.^[^ ^166^ ^]^ It was used as a capacitor dielectric with a temperature‐sensitive dielectric constant in implantable and bioresorbable temperature sensors.^[^ ^60^ ^]^ PBAT is a commercially available soft, flexible, and fully compostable plastic with excellent processing properties.^[^ ^167^ ^]^ It is an attractive candidate packaging material for wearable biodegradable sensors and its utility was demonstrated in the encapsulation of deformable temperature sensors.^[^ ^62^ ^]^ PBAT was also used as a matrix constituent of the biodegradable conductive paste which shows remarkable mechanical properties (elongation up to 36.4% without fracturing and bendability with retained conductivity up to 3.7 mm^−1^ of curvature).^[^ ^47^ ^]^

Finally, an interesting class of polymers for biodegradable sensors, which typically does not satisfy the traditional standards of degradability, are some conductive polymers like poly(pyrrole) (PPy),^[^ ^73^, ^168^ ^]^ poly(aniline) (PANI),^[^ ^127^ ^]^ and poly(3,4‐ethylenedioxythiophene):poly(styrene sulfonate) (PEDOT:PSS).^[^ ^152^ ^]^ These materials are thus usually chemically modified or combined with biodegradable polymer matrices to improve biocompatibility and degradation properties.^[^ ^169^ ^]^

### 2.4 Natural or Bioderived Materials

Degradable natural materials are an interesting alternative option for constructing biodegradable sensors. Among them, the dominant classes are carbohydrate polymers and polypeptides, both of hydrophilic nature. However, natural waxes recently emerged as hydrophobic materials offering moderate transient times and improved water resistance. Table 3 shows key properties of natural and bioderived polymers with established use in biodegradable sensing devices, such as cellulose‐based materials, silk, gelatin, chitosan, and natural wax. First, we focus on describing these materials in more detail. For instance, a crucial cellulose‐based material for biodegradable sensors is sodium carboxymethyl cellulose (Na‐CMC), which is a water‐soluble derivative obtained by etherification of cellulose (the most abundant polymer of glucose in nature).^[^ ^170^, ^171^, ^172^ ^]^ This material is a biodegradable polyelectrolyte able to form hydrogels with swelling behavior dependent on pH and ionic strength.^[^ ^172^ ^]^ Na‐CMC substrates typically decompose quickly in an aqueous environment (within a few min) and have found utility in the fabrication of bioresorbable printed circuit boards^[^ ^150^ ^]^ and the printing of bioresorbable conductors.^[^ ^68^, ^69^, ^70^ ^]^ In addition, Na‐CMC ink has been used to construct biodegradable capacitance‐based humidity sensors with extremely high sensitivity.^[^ ^120^ ^]^

Next, silk is a natural fibrous biopolymer produced by larvae of the silkworm *Bombyx Mori*, containing assembled polypeptide chains of fibroin and sericin. It has excellent biocompatibility supported by a long history of applications in surgical sutures, wound healing, and tissue engineering.^[^ ^173^ ^]^ Silk can be considered a fully resorbable material, for which the rate of degradation can be engineered by tuning the structure, crystallinity, porosity, and molecular weight distribution.^[^ ^174^, ^175^, ^176^ ^]^ As a sensor component, silk exhibits transparency, flexibility, and outstanding mechanical robustness, while being easily processable under ambient conditions.^[^ ^17^, ^177^ ^]^ In addition, silk can show interesting piezoelectric^[^ ^178^ ^]^ and triboelectric^[^ ^179^, ^180^ ^]^ properties. The synergy of these favorable properties has driven the use of silk as a substrate, encapsulant, and active component in different flexible biodegradable devices.^[^ ^100^, ^181^, ^182^ ^]^

Gelatin is a natural highly biocompatible, biodegradable, and cost‐effective polymer derived from the hydrolytic degradation of collagen with many established applications in the food and healthcare sectors.^[^ ^183^, ^184^ ^]^ An aqueous solution of gelatin can yield thermally reversible physically cross‐linked gels upon cooling below 35 °C.^[^ ^185^ ^]^ However, pure gelatin hydrogels are not stable under physiological conditions, and chemical or enzymatic cross‐linking methods are typically preferred to increase hydrogel stability and control its degradation.^[^ ^186^ ^]^ The introduction of additives such as glycerol, citric acid, and sugars can also significantly improve the thermodynamic stability, gelation, and mechanical properties of gelatin‐based gels, while also keeping good biodegradation ability (degradation period ranging from days to weeks).^[^ ^67^ ^]^ By engineering gelatin‐based gels at the molecular and structural level, it was possible to develop appealing biodegradable components for pressure, strain, temperature, and humidity sensing.^[^ ^39^, ^67^ ^]^ Composites of cross‐linked gelatin, incorporating glycerol and caprylic acid, were successfully applied for impedimetric monitoring of proteolytic degradation,^[^ ^187^ ^]^ while the composites of cross‐linked gelatin mixed with gum acacia enabled the encapsulation of microdroplet lasers with temperature‐sensitive emission wavelength.^[^ ^188^ ^]^

Chitosan is a linear polysaccharide derived by chitin deacetylation which dissolves in acidic aqueous solutions through the protonation of its primary amine groups.^[^ ^189^ ^]^ It is a versatile biomaterial with many desirable properties^[^ ^190^ ^]^ including biodegradability, biocompatibility, and antibacterial activity, which led to its broad use in biomedical products.^[^ ^191^ ^]^ Two key parameters determining the biological behavior of chitosan are molecular weight and deacetylation degree.^[^ ^192^ ^]^ By tuning these material properties and selecting appropriate fabrication methods, the duration of chitosan implant degradation can be adjusted to last between days and months.^[^ ^190^ ^]^ In biodegradable sensors, chitosan matrices proved useful in supporting the assembly of organic crystals with piezoelectric properties^[^ ^36^, ^193^ ^]^ suitable for pressure sensors. In addition, chitosan copolymerized with PVA (using phytic acid as a crosslinker) forms the backbone for ionically conductive hydrogels used in wearable strain sensors.^[^ ^164^ ^]^

Natural waxes are complex mixtures of different organic compounds including long‐chained polyesters, fatty acids, anhydrides, resins, and some fraction of short‐chained hydrocarbons.^[^ ^194^, ^195^ ^]^ They are attractive candidate materials for encapsulants and sealants in biodegradable sensors due to their hydrophobic nature limiting water uptake, while simultaneously containing several unsaturated sites and reactive functional groups enabling the tuning of degradation rates to reach full dissolution within a period ranging from days to weeks.^[^ ^37^ ^]^ Out of several types of natural waxes studied by Won et al.,^[^ ^37^ ^]^ candelilla wax had the most pronounced hydrophobic properties leading to limited water penetration and encapsulation degradation times of a couple of weeks for the 300‐µm‐thick sealing layer, presumably via the hydrolysis pathway proceeding through chain scission of reactive groups (e.g., esters and anhydrides) in its composition.^[^ ^194^ ^]^ In the same study, candelilla wax successfully served as a matrix for a conductive and biodegradable composite paste with W microparticles (C‐wax). Candelilla wax‐based materials served as components for sealing and electrically conductive interconnects in sensing systems for the measurement of pressure^[^ ^59^, ^64^ ^]^ and temperature,^[^ ^60^ ^]^ as well as in the realization of a degradable LED.^[^ ^21^ ^]^

Another group of relevant materials is natural polysaccharides with emerging applications in biodegradable sensors, including alginate, agarose, starch, levan polysaccharide, K‐carrageenan, and galactomannan. **Table** **4** showcases the key properties and uses of these materials. We also summarize their main characteristics in further text.

**Table 4:** Properties of natural polysaccharides with emerging applications in biodegradable sensors.

Material	Functional Properties	Applications	Degradation Medium	Degradation Time
Alginate	Biocompatible; Tunable degradation;	Electrolyte separator in batteries	PBS (pH 7.4, 37 °C)	≈9 days^[^ ^19^ ^]^
Agarose	Biocompatible; Slow degradation	Gel electrolytes in supercapacitors	PBS (pH 12, 65 °C)	≈24 h^[^ ^80^ ^]^
Starch (with high amylose content)	Flexible and stretchable; Tunable ionic conductivity and strain sensitivity	Strain and pressure sensors; Component of transient batteries	Nutritional soil	40 days (≈85% mass loss)^[^ ^196^ ^]^
Levan polysaccharide	Transparent; Flexible; Tunable degradation; Compatible with transfer printing	Substrate; Enclosure	In vivo	20 days^[^ ^40^ ^]^
K‐carrageenan	Flexible; Porous; Hydrophilic	Encapsulation	DI water, 90 °C	<5 min^[^ ^127^ ^]^
Galactomannan	Facile extraction; Resistant to organic solvents; Transparent; Compatible with metal deposition	Substrate for wearable and disposable sensors	Water, room temperature	5 min^[^ ^41^ ^]^

Alginate is a natural polysaccharide biologically derived from brown algae commonly used in the form of a cross‐linked hydrogel.^[^ ^197^ ^]^ The cross‐linking of alginate hydrogels is typically achieved rapidly under mild aqueous conditions in the presence of bivalent cations (e.g., Ca^2+^).^[^ ^198^ ^]^ These hydrogels are attractive for the fabrication of degradable sensing devices due to their cost‐effectiveness, excellent biocompatibility, and biodegradability.^[^ ^199^ ^]^ To achieve and control alginate degradation under physiological conditions, chemical modifications are typically applied in the form of partial oxidation and tuning of molecular weight distribution.^[^ ^197^, ^200^ ^]^ Degradation of partially oxidized alginate can occur even within days,^[^ ^19^ ^]^ but its degradation rate can be also slower and dependent on medium parameters (pH and temperature).^[^ ^201^ ^]^ Cross‐linked alginate hydrogels are increasingly used as polymeric electrolyte separators in biodegradable batteries.^[^ ^19^, ^65^ ^]^

Similar to alginate, agarose is a heteropolysaccharide extracted from red algae commonly applied as a gel in molecular biology for the separation of biomolecules using electrophoresis.^[^ ^202^ ^]^ It is well‐known as a biocompatible and biodegradable material in its hydrogel form.^[^ ^203^, ^204^, ^205^ ^]^ Agarose undergoes gelation at ambient temperature and the resulting hydrogels typically exhibit slow degradation rates,^[^ ^204^ ^]^ except in the presence of specific enzymes such as agarase^[^ ^206^ ^]^ or non‐physiological conditions in the aqueous medium^[^ ^80^ ^]^ (elevated pH and temperature). Because of the properties of the agarose hydrogel matrix, agarose is suitable for ion transport and is typically used as a gel electrolyte in flexible biodegradable supercapacitors.^[^ ^80^, ^207^ ^]^

Starch, a natural carbohydrate polymer, is known for its biocompatibility and biodegradability, particularly in the form of hydrogel films. Cost‐effective and easily processed, starch‐based hydrogels enriched with inorganic ions have emerged as appealing conductive materials for biodegradable sensors and electronics, provided that their mechanical properties are unaffected by water content.^[^ ^208^ ^]^ Achieving this requires increasing the content of amylose which is composed of linear chains, and imparts starch‐based hydrogels with good flexibility and stretchability.^[^ ^209^, ^210^ ^]^ Within starch‐based hydrogel films with high amylose content, dissociated ions from inorganic salts can serve as plasticizers and charge carriers, contributing to ionic conductivity and strain‐sensitive properties.^[^ ^211^, ^212^ ^]^ Tailored starch‐hydrogel‐based films demonstrated great potential as active components for transient batteries or self‐powered strain and pressure sensors.^[^ ^196^ ^]^

Levan is a natural fructan polysaccharide synthesized by many microorganisms and several plant species starting from sucrose as a precursor.^[^ ^213^ ^]^ It is capable of forming water‐soluble, flexible, and transparent thin substrates (≈200 µm) which can serve as promising transient substrates or enclosures in biodegradable sensors as demonstrated by Kwon et al.^[^ ^40^ ^]^ The authors showed that levan films can completely dissolve under physiological conditions in vitro after several hours and fully resorb in vivo after 20 days without notable harmful effects. The dissolution rate could be tuned by increasing the molecular weight or enhancing the cross‐linking degree through the addition of malic acid. Finally, the levan films showed compatibility with transfer printing and supported on‐demand programmable transience of Mg‐based components activated by exposure to NIR radiation (808 nm, power densities ≈W cm^−2^).

Among biologically derived polysaccharides, additional key materials for biodegradable sensors are K‐carrageenan emerging as an encapsulation material,^[^ ^127^ ^]^ and galactomannan emerging as a versatile substrate with favorable extraction and processing properties.^[^ ^41^ ^]^

Besides the aforementioned polypeptides, which are commonly used in biodegradable sensors, it is worth mentioning the protein bovine serum albumin (BSA) and amino acid glycine as interesting natural materials for biodegradable sensors, although still with limited use. BSA belongs to the group of most abundant proteins in the blood plasma of mammals.^[^ ^214^ ^]^ It is well‐known for its ability to bind versatile ligands, and it can act as a microscale carrier transporting different functional molecules in healthcare applications.^[^ ^214^, ^215^ ^]^ These beneficial properties of BSA were exploited to formulate white light‐emitting nanoparticles for pH sensing and probing of the intracellular environment.^[^ ^216^ ^]^ Glycine is a water‐soluble nonessential amino acid with a simple structure (containing only a single hydrogen atom in the side chain) that plays an important role in human health and metabolism.^[^ ^217^ ^]^ Under ambient conditions, glycine can crystallize into different (α, β, and γ) polymorphs.^[^ ^36^, ^218^ ^]^ Structures of β‐ and γ‐glycine are acentric and they show piezoelectric properties.^[^ ^219^ ^]^ A particularly interesting phase is β‐glycine which shows a high shear piezoelectric coefficient comparable to piezoceramics (178 pm V^−1^)^[^ ^220^ ^]^ and also exhibits ferroelectricity^[^ ^221^, ^222^ ^]^ unlocking the potential for more uniform polarization at larger scales. Due to this interesting combination of properties, glycine became an attractive candidate material for biodegradable pressure sensors.^[^ ^36^ ^]^

Several natural and biologically derived materials offer readily available, biocompatible, and cost‐effective options for the design of biodegradable sensors. However, these materials also pose some challenges in terms of manufacturing reproducibility compared to their degradable synthetic counterparts. Variations within source materials and limited compatibility with available processing methods make it difficult to obtain consistent material properties of the final products or components. With the advancement of mild processing methods and controlled production of source materials, natural materials, and their derivatives will be poised to play an increasing role in sustainable biodegradable sensing devices.

Although the field of biodegradable sensors is still in its infancy, many researchers managed to overcome the current challenges of material selection through intelligent approaches in sensor design, fabrication, and combining diverse material classes within a single device. Advances in materials science and engineering have enabled the development of fully biodegradable essential components for sensing devices, such as dedicated sensing elements, conductive traces, interconnects, insulating enclosures, power sources, and antennae for wireless communication. New materials for fully degradable electronic and optical components are an active area of ongoing research, holding the promise of developing degradable sensors fully integrated with the supporting electronics and photonics required for signal readout and transmission. To integrate highly diverse materials, which are often compatible with different processing methods into a functional sensor, various fabrication techniques need to be carefully and synergistically adapted for specific biodegradable materials. Many innovative fabrication approaches are also continuously developed to overcome still unresolved challenges encountered when manufacturing biodegradable sensors. In the following, we review the key fabrication techniques pertinent to biodegradable sensing devices.

## 3 Fabrication Techniques

**Scheme 2:**
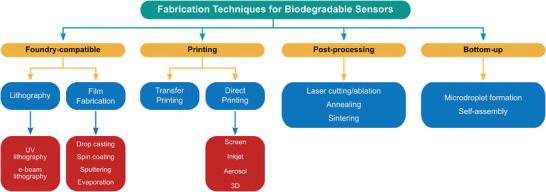
Overview and classification of key techniques used for the fabrication of biodegradable sensors.

A multitude of components is required to create a fully functional sensing system. Biodegradable sensors for medical applications should include components capable of measuring physiological parameters such as temperature, pressure, strain, pH, and concentration of specific molecules, or e.g., recording electrophysiological signals. Thus, sensor components, such as transducers converting signals from the sensing element to the measurable readout, interconnects, communication units transmitting the data outside of the body, acquisition systems, energy sources powering the sensor, supporting electronics, and encapsulation protecting the device from degradation, are all of critical importance. For implanted sensors, controlled rates of resorption and elimination are essential, thereby adding further complexity to the system. To achieve adequate medical‐grade performance of biodegradable sensors within the projected timeframe, fabrication techniques must be optimized and adapted to meet the often‐contradictory demands of sensor functionality and degradability. **Scheme** **2** provides an overview of key fabrication techniques used to construct biodegradable sensors, including the traditional foundry‐compatible methods known from semiconductor processing (e.g., spin coating, lithography, sputtering, etc.) as well as diverse printing, post‐processing, and bottom‐up methods adapted for the fabrication processes involving biodegradable materials.

**Figure 2:**
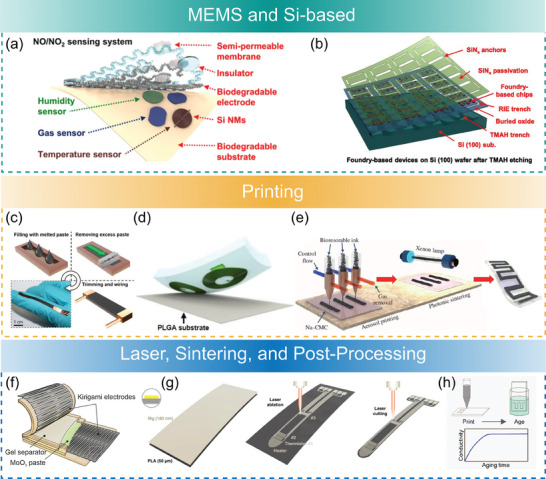
Examples of fabrication techniques for biodegradable sensors: a) A biodegradable, flexible silicon‐based electronic system that detects NO_x_ species with a record‐breaking sensitivity of 136 Rs (5 ppm, NO_2_). Reproduced with permission.^[^ ^107^ ^]^ Copyright 2020, The Author(s). b) Wafer‐scale release of foundry‐based, ultrathin silicon components for transient electronics. Reproduced with permission.^[^ ^101^ ^]^ Copyright 2017, National Academy of Sciences. c) Conductive patterns on biodegradable substrates obtained using screen printing. Reproduced with permission.^[^ ^83^ ^]^ Copyright 2023, The Authors. d) Mg RF coil being transferred from temporary polydimethylsiloxane (PDMS) substrate onto a biodegradable PLGA substrate. Reproduced with permission.^[^ ^23^ ^]^ Copyright 2019, WILEY‐VCH Verlag GmbH & Co. KGaA, Weinheim. e) Bioresorbable electronic patterns using aerosol printing and photonic sintering. Reproduced with permission.^[^ ^71^ ^]^ Copyright 2018, Science China Press and Springer‐Verlag GmbH Germany, part of Springer Nature. f) Kirigami‐patterned electrodes for stretchable and biodegradable batteries. Reproduced with permission.^[^ ^65^ ^]^ Copyright 2022, The Authors. g) Schematic for fabrication of microvascular flow sensing probes using laser ablation. Reproduced with permission.^[^ ^223^ ^]^ Copyright 2022, The Author(s). h) Enhancement of conductivity of PLA‐based ink through solvent aging. Reproduced with permission.^[^ ^82^ ^]^ Copyright 2020, American Chemical Society.

The methodologies used to process degradable materials are further summarized in **Figure** **2** with an emphasis on prominent application examples. Biodegradable materials are particularly challenging to process using conventional micro‐ and nanofabrication techniques because of associated process steps involving high temperatures, vacuum conditions, and exposure to various solvents including water. Although the adaptation of certain process steps enables working with various biodegradable materials, the range of materials processable with micro‐ and nano‐fabrication techniques remains limited because of the risks associated with thermal or chemical degradation during fabrication. The conventional techniques should be adapted to become compatible with more fragile degradable materials by avoiding specific steps (e.g., etching or annealing) or performing them under milder conditions. In parallel, the use of printing techniques such as transfer printing,^[^ ^23^ ^]^ inkjet printing,^[^ ^224^ ^]^ and screen printing^[^ ^150^ ^]^ has been extensively researched. Post‐processing methods including ink curing,^[^ ^225^ ^]^ solvent aging,^[^ ^82^ ^]^ and sintering^[^ ^68^ ^]^ have been also explored to improve the electrical and mechanical characteristics of printed structures.

### 3.1 Micro‐electromechanical Systems (MEMS)‐based Fabrication Techniques

The most common biodegradable material in conventional micro‐ or nano‐fabrication is Si, for which many fabrication techniques were initially established during MEMS development. Thinning down of Si using processes such as repeated cycles of oxide formation at elevated temperatures and removal of the thermally grown oxides using wet etchants such as hydrofluoric acid (HF) can be used to form Si nanomembranes (Si‐NMs). Implanted Si NMs are dissolvable in vivo and enable the use of established Si integrated circuit (IC) technology to fabricate highly conductive and biodegradable electronics.^[^ ^100^ ^]^ A biodegradable, flexible silicon‐based electronic system that detects NO_x_ species with a record‐breaking sensitivity of 136 Rs (5 ppm, NO_2_) reported by Ko et al. (see Figure 2a) was fabricated using such processes.^[^ ^107^ ^]^ Single‐crystal silicon nanomembranes (SC‐Si NMs) on silicon‐on‐insulator (SOI) wafers were thinned down to desired thickness (≈100 nm) for biodegradability, doped with phosphorous for electrical contacts, and released from the SOI wafer by undercut wet etching of the buried oxide (BOX) layer to transfer print the SC‐Si NMs onto a polymethyl methacrylate (PMMA)/diluted polyimide (PI) coated temporary substrate. After the formation of electrodes and insulators through standard photolithography, electron‐beam evaporation, and etching, the resulting components were transferred onto biodegradable substrates by removing the bottom PI layer. Although Si‐NMs are quite attractive for biodegradable devices, they also pose potential risks of hydrogen accumulation in the body due to hydrolysis.^[^ ^25^ ^]^ Alternatively, biodegradable germanium nanomembranes (Ge‐NM) can theoretically eliminate gaseous products during dissolution and exhibit a small forbidden width and large carrier mobility, thereby being relevant to high‐frequency devices and rapid data acquisition.^[^ ^112^ ^]^ Photolithography, inductively coupled plasma‐reactive ion etching (ICP‐RIE), buried oxide etching, and transfer printing using polydimethylsiloxane (PDMS) stamps onto a biodegradable substrate allow the fabrication of germanium‐on‐insulator (GOI) sensors (thickness: 200 nm Ge and 2 µm buried oxide) that can successfully distinguish the cross‐talk of different physiological signals such as temperature and strain, suggesting the feasibility of multi‐parameter biosensing.^[^ ^112^ ^]^

Some of the most important micro‐ and nanofabrication steps are discussed in more detail in the following sections.

### 3.2 Film Deposition and Conductive Layers

Films can be employed to provide structural or functional properties to developed sensors, and they can either be used as sacrificial or masking layers during the process of microfabrication or as protection of the base material from etching. In some devices, films can be used as electrical components. One approach is to exploit shadow masking, where a material, e.g., silk, can be used as a substrate on top of which an array of transient transistors and mechanical energy harvesters are directly deposited by using e‐beam evaporation or sputtered using high‐resolution stencil masks.^[^ ^95^ ^]^ Li et al. reported a flexible, biologically degradable, and wirelessly operated electrochemical sensor for real‐time NO detection with a low detection limit (3.97 nmol), wide sensing range (0.01 µM), and desirable anti‐interference characteristics. The device consisted of a bioresorbable substrate which is drop‐casted on frosted glass PLLA and poly(trimethylene carbonate) (PLLA–PTMC), thickness: 400 µm), sputtered ultrathin gold (Au) nanomembrane electrodes (thickness: ≈32 nm), and electrochemically deposited biocompatible poly(eugenol) film (thickness: ≈16nm) as the selective membrane.^[^ ^226^ ^]^ Apart from the traditional evaporation or sputtering of conductive layers, laser‐cut uniform foils of Mo (thickness: 15 or 25 µm) have been suggested as suitable electrode material for bioresorbable electrotherapy systems, where changes in impedance can be utilized to monitor the healing process at chronic wound sites.^[^ ^8^ ^]^

### 3.3 Lithography

(Photo)Lithography is an integral part of micro‐ or nanofabrication, which involves the imprint of a desired pattern onto a (photo) sensitive “resist” layer. In photolithography, the photoresist is exposed to UV light through a photomask having the desired pattern. The exposed areas are then selectively kept or removed using a developer solution depending on whether the photoresist is of negative or positive type. In the case of electron beam lithography (EBL), the resist layer is sensitive to electrons and can be exposed by an electron beam in a maskless manner. However, resist removal in both cases generally involves exposure to organic solvents and ultrasonication. Such processing may be incompatible with biodegradable substrates, and adaptations of corresponding steps are often necessary.

Researchers have reported the use of aqueous silk fibroin (SF) spin‐coated onto a substrate to act as either positive or negative resist for EBL, which can then be developed in water. Utilizing this all‐water‐based approach, the authors report the fabrication of nanoscale lithographic patterns from silk, including modifications with quantum dots, green fluorescent proteins (GFP), or enzymes (horseradish peroxidase).^[^ ^227^ ^]^ Silk has also been reported as a water‐based positive‐tone resist for photolithography using an argon fluoride (ArF) excimer laser operating at a wavelength of 193 nm.^[^ ^228^ ^]^ A more recent approach involves the use of a water‐soluble sacrificial layer of dextran spin‐coated onto a silicon substrate.^[^ ^229^ ^]^ A 15% (w/w) solution of PLA in chloroform is spin‐coated on top to form a 7 µm thick layer, followed by Ge deposition using an electron beam evaporator (thickness: 60 nm) to form a biodegradable protective layer. Subsequently, SiO_2_ (thickness: 80 nm) is sputtered as an adhesion layer for subsequent metallization. This sequence of steps allows to proceed with conventional lithographic processing without damaging the biodegradable polymer layer. Finally, the completed chip is submerged in DI water to release the structures from the substrate. As an alternative approach, transfer printing allows the use of established micro‐fabrication steps and the eventual “transfer” of the produced components onto a biodegradable substrate, as described in the following section.

### 3.4 Printing

#### 3.4.1 Transfer Printing

In transfer printing, a sacrificial substrate layer is used along with IC fabrication techniques. For example, the intended structures and layers of the desired device can be fabricated on a handling wafer using photolithography or other structuring methods, including a sacrificial layer such as PMMA. Diluted PI can be used as a protective layer to prevent device exposure to water. A selective solvent is then used to remove the sacrificial layer so that the device can be picked up using a PDMS stamp and transferred onto a target substrate. This enables the use of biodegradable materials for the target substrate, since otherwise, the chosen substrate material may not survive exposure to harsh treatments and water during previous fabrication steps. Finally, the protective layer is removed.

Foundry‐compatible and wafer‐scale biodegradable electronics (Figure 2b) have also been demonstrated using transfer printing methods.^[^ ^101^ ^]^ Controlled release of fully formed circuits and/or circuit components on a standard 6‐inch SOI wafer is achieved through anisotropic wet chemical etching, followed by integration onto a biodegradable target substrate. SiN_x_ and BOX layers on the top and bottom side, respectively, protect the device blocks from the etchants, while nitride anchors keep the resulting free‐standing blocks tethered to their original locations. This approach could potentially pave the way for mass production of commercially available biodegradable sensors.

Further research suggests that eco‐ or bioresorbable versions of all major classes of MEMS devices, including electrocapacitive sensors, electrostatic actuators, and electrothermal actuators can be realized using similar transfer printing approaches by utilizing trilayers of Mo/SiO_2_/W for electrical connections, bioresorbable substrates such as a polyanhydride‐based polymer (PAP, thickness: 100 µm), and appropriate encapsulations.^[^ ^230^ ^]^ In this case, bioresorbable natural waxes can be used as water barriers whereas tissue‐like hydrogel adhesive matrix (HAM) of covalently crosslinked bifunctional PEG glycol–polylactide–diacrylate (PEG‐LA‐DA) macromers and ionically crosslinked sodium alginate enables robust adhesion to biological tissues, diffusion of proteins or molecules for sensing, and confinement of device fragments from fracture or partial disintegration through dissolution. In general, due to fluid motion, the aqueous etchants used in the case of wet transfer printing methods can distort the shapes of device patterns through flotation, making it challenging to precisely control the retrieval of devices from the initial or temporary substrates and subsequent alignment onto the target substrates.^[^ ^231^ ^]^ Certain dry transfer printing approaches, e.g., using weak adhesion between PDMS and PI^[^ ^232^ ^]^ or based on laser irradiation^[^ ^233^ ^]^ have been also explored. Nevertheless, transfer printing remains associated with high cost, sophisticated equipment, and long fabrication times. Hence, direct printing methods are being widely researched, and we will discuss them in the next section.

#### 3.4.2 Direct Printing

Processes involving an extensive number of steps including lithography, deposition, lift‐off, and/or etching are generally time‐consuming as well as wasteful in terms of resources. Various direct printing techniques allow rapid, cost‐effective, high‐yield, and low‐temperature processes that are compatible with the fabrication of biodegradable and flexible electronic devices.^[^ ^234^ ^]^ The most commonly used techniques are discussed below.

*Screen Printing*: Screen printing is the most mature direct printing technique used in conventional electronics for the printing of metallic structures (e.g., interconnects in printed circuit boards), which can be repurposed for transient sensing devices. It is a technique where a substrate is covered with a protective stencil only leaving selective areas exposed. A mesh is then used to transfer conductive ink and a slide is moved across the screen to fill open areas of the stencil with transferred ink (Figure 2c). Thus, water‐soluble biodegradable materials may also be applied with this technique. An example is the fabrication of bioresorbable electronics via stencil‐based screen printing of Zn NPs on biodegradable substrates to create conductive patterns.^[^ ^68^, ^70^ ^]^ To prepare Zn NP ink for screen printing, Zn NPs are evenly dispersed in polyvinylpyrrolidone (PVP)‐containing solvent. Biocompatible and biodegradable patches of E‐skin based on an aerogel of gelatin methacryloyl and screen printing of conductive ink have been reported to simultaneously monitor body temperature, hydration, and biopotentials via electrophysiological sensors, as well as detect glucose, lactate, and alcohol levels via electrochemical sensors.^[^ ^235^ ^]^ Researchers have explored various inks and pastes for screen‐printing purposes. An interesting point to note is the effect of dispersing agents, which enhance the mechanical properties of the ink or paste.^[^ ^47^, ^83^ ^]^ A highly conductive and mechanically stable bioresorbable W paste is reported, achieved through a thermoplastic beeswax matrix and glycofurol as a W dispersion agent.^[^ ^83^ ^]^ Although screen printing is a fast and simple process, the resolution of stencils may restrict the construction of complex layouts with very small feature sizes.

*Inkjet and Aerosol Printing*: Inkjet printing is a direct writing technology, that uses droplets of ink on a variety of substrates without the need for any mask. Inkjet printing demonstrates compatibility with various biodegradable materials for creating transient microelectronic devices containing conductor, dielectric, and semiconductor functional layers.^[^ ^224^ ^]^ However, the inkjet technique suffers from limitations in minimum feature size, low stability of metallic inks, narrow tolerances regarding viscosity, and insufficient repeatability for batch fabrication. Results from the study by Mahajan et al.,^[^ ^71^ ^]^ indicate that aerosol printing and photonic sintering of Zn NPs on Na‐CMC substrates can potentially yield mass fabrication of bioresorbable electronics. Compared to inkjet printing, aerosol printing can tolerate a large viscosity range and support a wider choice of materials. In aerosol printing, a nebulizer atomizes the ink which contains, e.g., bioresorbable Zn NPs, into droplets of 1 µm in diameter through Argon (Ar) gas flow supplied by the flow controllers. This results in an aerosol mist that contains both ink droplets and Ar molecules. The printed pattern is cured at a temperature lower than 100 °C to remove the solvent, and curing is then followed by photonic sintering with pulsed xenon light (Figure 2e). By combining two cascaded sintering steps (flashlight and laser), the conductivity of the bioresorbable patterns was improved up to 34722.2 S/m. Aerosol printing, however, shares certain common challenges with inkjet printing, e.g., overspray, gelling, pooling, and cloudiness.^[^ ^236^ ^]^

*3D Printing*: Established top‐down (subtractive) methods using lithography, micromachining, etc. are generally time‐consuming and result in the wastage of resources. 3D printing is an additive manufacturing process in which a three‐dimensional object is printed, layer by layer, according to a digital computer‐aided design (CAD) through deposition, fusing, or solidification of the building materials. Traditionally, various polymers have been used as building components in 3D printing methods. With the advancement of knowledge about compatible materials, 3D printing is becoming increasingly important for the manufacturing of biodegradable sensors. The 3D printability of graphene−regenerated silk/tannin (G‐RS/T) solutions to fabricate multilayer grids was demonstrated by Chiesa et al.,^[^ ^237^ ^]^ The multifunctional composite of silk fibroin and plant‐derived polyphenol (chestnut tannin) modified with graphene nanoplatelets behaves as an adhesive on various substrates. Furthermore, silk fibroin exhibits piezoelectric properties. This enables the design of a bioresorbable 3D printed flexible and self‐adhesive piezoelectric device that could have applicability in monitoring the motility of the gastrointestinal tract and for the diagnosis of motility disorders. In another report by Park et al., a biodegradable polymeric stent from PCL has been 3D printed and combined with a micromachined pressure sensor.^[^ ^238^ ^]^ The polymer stent exhibited significant improvement in sensitivity to degradation as compared to a metal stent. A viscous PLA solution with high‐mass loading of W particles has been deposited from a rotary screw‐driven syringe‐needle assembly using the robotic material extrusion method to create conductive traces, the conductivity of which was then enhanced through solvent aging (Figure 2h).^[^ ^82^ ^]^ Furthermore, composites based on PGS, carbon nanotubes (CNTs), and salt microparticles have been used as inks to 3D print triboelectric nanogenerators (3DP‐TENGs) for physiological sensing and biomechanical energy harvesting.^[^ ^239^ ^]^ Although 3D printing technologies offer various advantages compared to traditional fabrication methods such as dip‐coating or spin‐coating of a liquid sensing layer on a substrate, traditional methods remain more cost‐effective. Hence, 3D printing technology is currently more suitable for small rapid prototyping and research, while traditional manufacturing is still more feasible for industrial production.^[^ ^240^ ^]^

### 3.5 Laser, Thermal, and Photonic Processing

Low‐cost fabrication of bioresorbable devices using screen printing methods generally involves biodegradable powders of Mg, Zn, or W. However, bioresorbable metallic materials such as Mg and Zn can readily form surface oxides and, combined with the effect of binders used to prepare the ink, the achievable conductivities of the pastes are significantly lower than those of bulk metals. Thermal instability of bioresorbable substrates poses additional challenges for sintering methods that employ overall or intensive localized heating. Thus, improvements in printing and sintering techniques are important aspects to investigate.

Mechanical annealing has been shown to enhance the target properties of certain materials. Applying a compressive pressure of 250 bars to biocompatible amino acid crystal powders (with ≈13% residual water) can produce compact films with up to 12 times the piezoelectric constant as compared to the untreated version.^[^ ^241^ ^]^ Such mechanically annealed crystal films also exhibit flat and smooth surfaces, thereby facilitating improved contact with electrodes and resulting in higher output voltages. An evaporation–condensation‐mediated laser printing and sintering method^[^ ^69^ ^]^ was reported by Shou et al. for low‐cost manufacturing of bioresorbable conductors using Zn NPs. A transparent glass was coated with a Zn NP suspension and dried to form a 2.5 µm thick film. It was then gently pressed onto a Na‐CMC bioresorbable substrate with the NPs facing the substrate. Zn traces with high crystallinity were directly printed on the receiving Na‐CMC by scanning a continuous‐wave (CW) fiber laser through the top glass slide. Zn traces with linewidth ca. 40 µm and sub‐µm thicknesses were fabricated in the process. Another laser sintering technique to weld naturally oxidized Zn microparticles into biodegradable conductors was reported by the group of Feng.^[^ ^68^ ^]^ PVP was dissolved in ethanol at 20 wt% and then mixed with Zn microparticles at a weight ratio of 25:4. The mixture was homogenized at 2000 rpm for 3 min to yield a viscous slurry. The Zn composite was printed onto biodegradable substrates using stainless steel stencil masks on a manual screen‐printing machine. After natural drying for 30 min, printed patterns of 52.5±0.4 µm thickness were obtained. A benchtop laser marking system equipped with a pulsed UV laser source at 355 nm wavelength and 1.1 W maximum output power was used to perform the laser sintering. In comparison to the as‐printed composites, the corresponding conductivity showed an increase by nine orders of magnitude.

Transparent biodegradable polymer substrates allow for selective sintering of NPs using a photonic sintering method, while the substrate is kept unaffected. Highly conductive traces for printable bioresorbable electronics have been demonstrated with inks containing mechanically milled irregular Zn NPs subjected to the photonic sintering effect at room temperature using a pulsed xenon lamp.^[^ ^70^ ^]^ Alternatively, laser ablation can be used to pattern metals on a polymeric or other biodegradable substrate, avoiding the necessity to screen‐print or use wet etchants and traditional lithography steps for pattern transfer. For example, bioresorbable microvascular flow sensing probes have been fabricated through laser ablation used to pattern vacuum deposited Mg (thickness: 180 nm) on a PLA substrate (thickness: 50 µm), as shown in Figure 2g.^[^ ^223^ ^]^ The PLA substrate is then cut into a needle‐shaped structure via another ablation step. Replacement of a Mg layer with a multi‐layered stack of SiO_2_/Mg/SiO_2_ (thickness: 100/180/100 nm) can further improve performance stability when the sensor is exposed to biofluids. Such ablation techniques are maskless direct writing used for rapid prototyping. However, laser ablation and photonic sintering are associated with localized and instant heating, leaving temperature‐sensitive substrates susceptible to damage such as distortion or crack propagation.^[^ ^229^ ^]^ The underlying effects and physical changes brought about by bioresorbable nano‐ or microparticles require further systematic analysis as well. Furthermore, the ablation of metals generally requires expensive lasers with pico‐ or femtosecond pulse widths.^[^ ^242^ ^]^

### 3.6 Bottom‐Up Fabrication Approaches

Despite having electrodes and sensing areas at the microscale, biodegradable sensing systems relying on previously mentioned fabrication methods tend to remain at the macroscale. These sensing systems are associated with invasive insertion methods (for implanted sensors) and are affected by the common challenges regarding interconnection and power supply. Utilization of functional (nano)materials to assemble optically active microstructures can enable injectable alternatives and remote sensing.

Franklin et al.,^[^ ^188^ ^]^ reported injectable slurries of well‐defined microparticles, which operate as temperature‐sensitive photo‐pumped lasers. The microdroplet laser core comprises a cholesteryl ester compound and a uniformly dispersed organic fluorescent dye (Nile Red). The cholesteric liquid crystal (CLC) exhibits a periodic variation in refractive index radially throughout the droplet. The CLC microdroplets were encapsulated in shells formed by cross‐linked hydrogels of 50% porcine skin gelatin and 50% gum acacia via complex coacervation. The encapsulation stabilized the CLC and protected its core from the surrounding biological environment. Electrostatic interactions among the charged macromolecules mediated phase separation. The thickness of the resulting hydrogel can be adjusted by tuning the pH and initial concentrations of the copolymers. In the report, thicknesses of up to 30 µm were used. The lasing mechanism was solved through optical pumping of CLC microdroplets with a frequency‐doubled, Q‐switched neodymium‐doped yttrium‐aluminum‐garnet (Nd:YAG) laser operating at 532 nm. Ex vivo demonstrations in Casper fish illustrated that a sensing system using such microdroplet lasers can measure temperature within biological tissues with a sensitivity of 0.01 °C.

Another study by Heah et al.,^[^ ^38^ ^]^ demonstrated a method to self‐assemble SF, a naturally abundant protein, into spherical microparticles for use as optical resonators. The SF microspheres were prepared using a modified water‐on‐oil emulsification method using an aqueous dispersion of SF and Span80 as a non‐ionic surfactant. The resultant micelles were centrifuged and washed with ethanol to remove any remaining surfactant. A powdery sample of the SF microspheres was then dyed through dispersion in a DMF solution of AR52 for 6 h at 85 °C to harness the photoluminescent properties. The resulting dyed SF microspheres exhibited optical resonance and their diameter expanded proportionally to the surrounding humidity due to their hygroscopic nature. The red shift of resonant peaks was in linear relation to the surrounding humidity and the system was able to measure relative humidity over a wide range of up to 95%.

### 3.7 Fabrication Approaches for Supporting Sensor Components

Since complete sensing platforms require various relevant components such as power sources, interconnects, and communication systems, the fabrication of these biodegradable components is an important aspect of the research of biodegradable sensors. Bioresorbable batteries^[^ ^19^ ^]^ and supercapacitors,^[^ ^80^ ^]^ or wireless power transfer^[^ ^23^ ^]^ and communication^[^ ^22^ ^]^ systems can allow biodegradable sensors to function more independently in the absence of direct physical connection to the external analyzers or power sources.

Guo et. Al.^[^ ^23^ ^]^ reported a bioresorbable system where a Mg coil was used as the receiver on a PLGA substrate and a transmitter comprising a rotating magnet was used for wireless power transfer. A 30‐µm magnesium foil was patterned using photolithography and wet etching with diluted HCl while staying attached to a PDMS substrate for handling. After soaking the Mg coil in ethyl acetate for 1 min, the coil was transferred onto a 30 µm‐thick PLGA substrate (Figure 2d). The complete inductor coil was formed by placing two individual Mg coils face‐to‐face while having a thin PLGA dielectric interlayer and opening for interconnections. The transmitter was fabricated using a disc magnet integrated into an electric motor mounted in a copper fixture. Further enhancement in power transfer was achieved through the introduction of a magnetic field concentrator comprising a composite of PLGA and biodegradable iron oxide nanoparticles (≈50 nm in diameter) on top of the receiver coils.

A high‐performance fully biodegradable magnesium–molybdenum trioxide battery was reported by Huang et al.,^[^ ^19^ ^]^ where a foil of Mg was used as the anode while alginate cross‐linked by calcium ions was used as the electrolyte. A paste comprising the powders (MoO_3_ or Mo) and PLGA dissolved in acetone (65:35, Mw = 75 000) with a final ratio of 2 g/0.5 g/8 mL for the powder/PLGA/acetone respectively, was cast onto a 30 µm‐thin foil of Mo to form the cathode. MoO_3_ was chosen as it is soluble in aqueous solutions and demonstrates biocompatibility at a controlled level. Due to the permeability of PLGA to aqueous components in electrolytes, the 3D porous network of MoO_3_ formed by the paste increased the active surface area and enhanced battery performance (output voltage and current). When compared to a MoO_3_ thin film (≈1 µm) formed by magnetron sputtering (100 W, 5 Pa Ar), the MoO_3_ paste cathode increased to 1 V. The thickness of the MoO_3_ paste cathode influences the discharge behavior of the battery over time, with 350 µm thick films achieving stable voltages up to 1.6 V for 48 h or longer, and 200 µm thick films exceeding 1.45 V for more than 50 h. Meanwhile, the alginate hydrogel electrolyte and polyanhydride/PLGA encapsulation prolonged the operational lifetime of the battery.

Biodegradable microsupercapacitors (MSCs) were demonstrated using transient metals (such as W, Fe, and Mo) for electrodes, hydrogel electrolyte (NaCl/agarose gel), and a biodegradable PLGA substrate, encapsulated with polyanhydride films.^[^ ^80^ ^]^ The electrodes were fabricated using lithography, sputtering, and lift‐off on a handling wafer. The fabricated electrodes were then transferred using a PDMS stamp onto a PLGA film. To prepare the electrolyte, 1% (w/v) of agarose powder was added to NaCl solution (0.35 M) and mixed vigorously at 120 °C. After 20 min, the solution was poured onto a glass plate at room temperature to yield a solid gel. After cooling, this gel electrolyte was cut to a suitable size and removed from the glass plate. At a current density of 0.05 mA cm^−2^, the microsupercapacitors fabricated using W (300 nm), Fe (200 nm), and Mo (300 nm) demonstrated areal capacitances of 0.02, 0.18, and 0.61 mFcm^−2^, respectively.

Mo foils in combination with a readily deformable MoO_3_ paste and Mg foils were employed as high‐power density electrode materials in a stretchable and biodegradable battery using kirigami‐patterned electrodes.^[^ ^65^ ^]^ Mo and Mg metal foils were gently pressed onto an intrinsically elastic, degradable, and sticky PGS substrate (1 mm thickness). The foils were patterned using a Trotec Speedy 300 FLP‐laser to form Kirigami structures which enable biaxial stretchability while preserving electrode area and high electrochemical performance (Figure 2f). The heat generated or dissipated onto the soft gel below the cutting areas facilitated further bonding between the freshly cut foils and the soft gel, while the uncut gel ensured the electrodes stretched in a planar manner. The kirigami‐patterned foils on the PGS substrate did not show any significant resistance variation during strain cycling measurements (<50% regime) with repeated stretching to 20% of the Mo electrode showing no increase in resistance up to 25 000 cycles.

In addition to power sources such as batteries and supercapacitors, transient light‐emitting diodes (LEDs) that can completely dissolve in aqueous solutions have also been reported.^[^ ^21^ ^]^ Thin films of highly textured ZnO were grown on thinned Si (111) through a pulsed laser deposition (PLD) process (248 nm, KrF excimer laser, 25 ns pulse duration, 15 Hz, 200 mJ per pulse) and patterned using a combination of lithography and wet etching. PLD allows the growth of ZnO films under a broad range of oxygen background pressures (PO_2_), thereby enabling the modulation of the emission spectrum and conductivity through a controlled density of oxygen vacancies. SiO_2_ (≈15 nm thickness; 20 µm × 20 µm window as an active area) as a biodegradable insulation layer, uniform Mo film as a transparent, ultrathin (8 nm) electrode allowing escape of emitted light, and relatively thick W film (100 nm, also with a patterned window) as an electrical lead with low resistance path to the active area, were fabricated through subsequent photolithography, sputtering, and lift‐off steps. Mg wires (250 µm × 250 µm cross‐section) provided electrical connections to external power sources. The thin films (200 nm) of highly textured ZnO served as semiconductors for light generation.

### 3.8 Innovative Fabrication Approaches

The development of biodegradable sensors is generally constrained by the choice of compatible materials and the fabrication processes must consider the limitations posed by the constituent materials in terms of exposure to various solvents, humidity, high temperatures, vacuum, and other relevant processing parameters. Furthermore, depending on the intended function and placement of the sensors (e.g., on the skin or implanted within the host), additional aspects such as hemocompatibility, rate of degradation and elimination, and composition of final degradation products must be considered. The power consumption of the devices as well as the costs and complexity of the fabrication process also play a critical role in making the sensors commercially viable. Hence, a plethora of innovative fabrication techniques have been investigated to either achieve a specific quality or enhance the feasibility of manufacturing.

A biodegradable, breathable multilayer piezoresistive pressure sensor was reported by Liu et al.,^[^ ^126^ ^]^ where the inherent fractal structure of magnolia leaf veins and 3D porous hierarchical structure of the fabricated sensor led to a high‐pressure sensitivity of 6.33 kPa^−1^ over a wide range (0.03.6 kPa). Due to the interlacing of natural leaf vein isolation layers with those coated with conductive silver nanowires (Ag‐NW), the initial current of the device without any external load is very small and minimizes power consumption to a large extent. Mature magnolia leaves are treated with an alkaline hydrolysis process to obtain the leaf veins. PLGA and PVA nanofiber films (PLGA‐NF and PVA‐NF, respectively) are fabricated through electrospinning. Ag‐NWs are sprayed onto PVA‐NF and leaf veins. PLGA layers on top and bottom act as encapsulating layers, whereas leaf veins coated with Ag‐NW and pure natural leaf veins are interlaced into multilayer stacks as pressure‐sensitive layers. PVA‐NF films coated with Ag‐NW act as top and bottom electrodes. Overall, the reported wearable sensors are suitable for real‐time monitoring of human physiological signals and human‐machine interface applications.

A ferroelectric nanogenerator‐driven skin sensor based on edible porcine skin gelatin was demonstrated by Ghosh et al.,^[^ ^39^ ^]^ To induce polarization confinement and enhance the ferroelectric properties of gelatin, microstructured surfaces (e.g., microdome, micropyramid, and micropillar) were arranged to face each other in interlocked structures. To fabricate the micropatterned films, inverse PDMS molds were prefabricated using soft lithography on Si masters coated with FOTS (1H,1H,2H,2H perfluorooctyltrichlorosilane) as an anti‐adhesion layer. 30% w/v gelatine solution (type A, porcine skin, gel strength ≈300 g Bloom) was then spin‐coated (≈1000 rpm, 90 s) on an oxygen plasma treated (≈3 s) PDMS mold and dried at 40 °C for 24 h under different humidity conditions (40‐80%). After peeling off from the PDMS mold, the films were cross‐linked using glutaraldehyde (GA) solution (25% in H_2_O) under a saturated vapor pressure of GA in a vacuum chamber for 24 h. All microstructures had similar feature sizes, e.g., 8 µm diameter, 15 µm pitch, and 4 µm height. Mg deposited using DC sputtering on the planar side of the micropatterned gelatin films was used for biodegradable electrodes and the interlocked films were encapsulated by gelatin films using a spray bandage. The self‐powered device was capable of sensing temperature, pressure, and surface texture variations.

Since the operational lifetime of bioresorbable sensors is an important factor, prolonged periods of stable operation are targeted by researchers in many cases. Shin et al.,^[^ ^243^ ^]^ report a Si‐based fabrication method where thermally grown silicon dioxide (t‐SiO_2_) acts as a biofluid barrier and protects the bioresorbable sensors to promote stable operation over extended periods. A pair of device‐grade SOI wafers were bonded together using amorphous silica formed by PDMS calcination. The fabrication proceeded through the etching of silicon wafers using ICP‐DRIE, thinning of BOX layers using buffered oxide etchant (NH_4_F:HF = 6:1), and patterned exposure of Si membrane for the electrical contacts (using sputtered and wet etched Mo, ≈100 nm × 150 µm × 150 µm).

Biological processes in cells and even on a molecular scale can produce certain signals, which are too weak to detect using conventional macroscopic sensors. Nanowire‐based biosensors (for example using silicon^[^ ^244^, ^245^, ^246^ ^]^) offer high surface‐to‐volume ratios and high sensitivity, and these can be combined with the advantages of optical methods (for example, anti‐electromagnetic interference and multiple retrieval signals) to develop powerful sensing tools. Zhang et al.,^[^ ^115^ ^]^ developed nanowires based on PLA which were drawn directly to form optical waveguides. PLA is dissolved in dichloromethane and stirred to form a homogeneous solution. A tapered fiber is dipped into the solution and pulled out quickly, forming PLA nanowires that stick to the end of the tapered fiber due to the rapid evaporation of the solvent. Optimization of the PLA solution concentration and the drawing speed enables size control of the PLA nanowires. An evanescent coupling method using two tapered fibers enables light input and detection. The evanescent wave is sensitive to the environment and allows the sensing of ultralow concentrations of cytochrome c (cyt c) as well as real‐time monitoring of the apoptosis process of yeast cells. The PLA nanowires can also sense pH changes when doped with CdSe/ZnS quantum dots. Another study reports wireless, bioresorbable temperature sensors that exploit multilayer photonic cavities for continuous optical measurements of regional, deep‐tissue microenvironments.^[^ ^105^ ^]^ The sensor comprises a cavity defined by a distributed Bragg reflector (DBR) using 8 bilayers of SiOx/SiNy, a Fabry‐Perot cavity of monocrystalline Si, a defect layer of SiO_x_, and a biodegradable PLGA substrate. The design for this resonant cavity decouples the temperature sensing from other interfering factors such as the angle of light incidence, scattering in the tissue, and absorption. In vivo monitoring of subcutaneous temperature in mice demonstrated the potential applicability of these sensors in biomedical research and clinical diagnosis.

A broad range of established fabrication techniques has been adapted to process biodegradable materials and address the common challenges, such as sensitivity to processing at elevated temperatures and pressures and solvent incompatibility. These include traditional micro‐ and nano‐fabrication involving lithography, deposition of thin films, and transfer printing, as well as the plethora of more cost‐effective direct printing methods. Laser processing approaches coupled with bioinspired strategies and bottom‐up synthesis are also gaining increasing significance in the fabrication of biodegradable sensing devices. Due to the diversity of materials that are commonly employed to construct biodegradable sensors, several techniques must be used in synergy to produce a functional sensor. The selection of appropriate techniques is additionally determined by the required sensor design. In the following, we review the sensor design concepts for physical and chemical sensors, as well as their relationship with the employed sensing principles.

## 4 Physical Sensors: Design and Sensing Principles

Physical sensors can detect and quantify various body signals generated from human physiological activities and monitor human health status in real time. The physiological activities can be detected by physical sensors based on electrical or optical signals. The development of biodegradable physical sensor mechanisms based on capacitive, resistive, piezoelectric, and triboelectric effects has been extensively investigated to date. Sensing mechanisms based on optical properties, such as evanescent fields and optical cavity resonators, have also been gradually developed. We will introduce their design and sensing principles in this section.

### 4.1 Principles of Electrical Signal Detection

#### 4.1.1 Resistance

**Figure 3:**
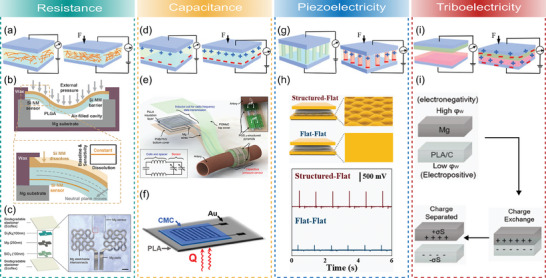
Electrical sensing principles of biodegradable physical sensors. a) Mechanism of piezoresistive sensing. Reproduced with permission.^[^ ^247^ ^]^ Copyright 2021, Wiley‐VCH GmbH. b) Patterned Si NM offers a piezoresistive response to bending strains that result from differences between the pressure of the surroundings and that of the air trapped inside the cavity. Reproduced with permission.^[^ ^64^ ^]^ Copyright 2020, WILEY‐VCH Verlag GmbH & Co. KGaA, Weinheim. c) Resistive temperature sensor based on the temperature dependence of thin‐film Mg. Reproduced with permission.^[^ ^62^ ^]^ Copyright 2017, WILEY‐VCH Verlag GmbH & Co. KGaA, Weinheim. d) Mechanism of piezocapacitive sensing. Reproduced with permission.^[^ ^247^ ^]^ Copyright 2021, Wiley‐VCH GmbH. e) Capacitive pressure sensor with an exposed view of the bilayer coil structure for wireless data transmission and the cuff‐type pulse sensor wrapped around the artery. Reproduced with permission.^[^ ^6^ ^]^ Copyright 2019, The Author(s), under exclusive license to Springer Nature Limited. f) The capacitance‐based carboxymethyl cellulose humidity sensor. Reproduced with permission.^[^ ^120^ ^]^ Copyright 2021, American Chemical Society. g) Mechanism of piezoelectric sensing. Reproduced with permission.^[^ ^247^ ^]^ Copyright 2021, Wiley‐VCH GmbH. h) Schematic illustration of assembly strategy and piezoelectric output voltage for the pressure sensor with flat and microstructured electrodes. Reproduced with permission.^[^ ^253^ ^]^ Copyright 2022, Elsevier Ltd. All rights reserved. i) Mechanism of triboelectric sensing. Reproduced with permission.^[^ ^247^ ^]^ Copyright 2021, Wiley‐VCH GmbH. j) Principle of the bioresorbable triboelectric sensor based on contact electrification and electrostatic induction. Reproduced with permission.^[^ ^63^ ^]^ Copyright 2021, Wiley‐VCH GmbH.

A resistance sensor can convert external stimuli into resistance change. The resistance of a material is calculated as *R* = *ρL*⁄*A*, where *ρ*, *L*, and *A* are the material resistivity, length, and cross‐sectional area, respectively.^[^ ^247^ ^]^ Based on all of the above parameters, some resistance‐based sensors can be designed. The piezoresistive effect is a change in resistance caused by the structural deformation of the material, and such change can be visualized by an electric signal (**Figure** **3**a,b).^[^ ^30^ ^]^ The change in resistance of piezoresistive material is proportional to its gauge factor.^[^ ^248^ ^]^ Piezoresistance‐based sensors are composed of conductive fillers and stretchable substrates and their inherent properties and microstructure determine the sensor performance.^[^ ^247^ ^]^ Generally, piezoresistive sensors exhibit a resistance that is proportional to the sum of their bulk resistance and contact resistance with their electrodes. Pressure (or strain) alterations change bulk resistance and contact resistance, which determines the device sensitivity. To achieve high sensitivity, microstructures and/or porous structures (e.g., sponges) are required, enabling large deformations and large resistance changes under applied pressure.^[^ ^64^, ^249^ ^]^

Resistance can also change in response to temperature variations. The electrical resistance of a thin metal film, e.g., Mg or Zn, shows temperature dependence. In conductive materials, higher temperatures cause electron vibrations which prevent the free flow of electrons, causing an increase in resistance.^[^ ^250^ ^]^ Salvatore et al. reported a fully biodegradable temperature sensor using Mg traces as the sensing element, which yields a resolution of 200 mK (Figure 3c).^[^ ^62^ ^]^

#### 4.1.2 Capacitance

Typically, capacitive sensors consist of a parallel plate configuration with an intermediate dielectric layer sandwiched between two electrodes (Figure 3d). The capacitance of the device is governed by the equation: *C* = *ε*_r_ *ε*_0_ *A*/*d*, where *C* is capacitance, *ε*_r_ *ε*_0_ is the dielectric constant of materials between two plates, *A* is the area of overlap of the two plates, and *d* is the separation distance between the plates. Various polymers with high elasticity and compressibility or deformability have been explored as dielectric layers. Micro‐ or nano‐fabrication techniques can improve the sensitivity and compressible space of the electrodes or dielectric layers by introducing different microstructures.^[^ ^6^, ^7^, ^30^, ^76^ ^]^

The temperature sensor can also be designed based on the temperature‐dependent dielectric constant of dielectric layers (e.g., PEG).^[^ ^60^ ^]^ Interdigitated electrodes (IDEs) are a popular structural pattern in the field of biosensors. IDE capacitance depends on the number of fingers, finger width, gap width, finger overlap, and thickness of the substrate, as well as the dielectric constant. As a result of these properties, humidity sensors are designed; water is absorbed into a humidity‐sensitive substrate and this changes the capacitance of the substrate (Figure 3f).^[^ ^120^, ^251^ ^]^

#### 4.1.3 Piezoelectric Effect

Piezoelectricity refers to the generation of an electrical charge in certain special classes of materials in response to mechanical stress or vibration. The piezoelectric effect is a result of an electric dipole moment and polarity in a solid. Piezoelectric sensors consist of two electrodes with vertical alignment and a piezoelectric material sandwiched between them. External pressure leads to the deformation of the piezoelectric material and results in spatial separation of positive and negative charges; such change can be monitored by an electric signal, e.g., open‐circuit voltage (*V*_oc_) and short‐circuit current (*I*_sc_),^[^ ^39^ ^]^ between two electrodes (Figure 3g,h). Piezoelectric sensors have high sensitivity and fast response time. Thus, they are widely used in the detection of dynamic pressures, but piezoelectric sensors are not well‐suited to sense static pressures or loads because the piezoelectric effect is transient in nature.^[^ ^252^ ^]^ Micro‐ and nano‐fabrication methodologies are introduced to endow electrodes or piezoelectric layers with various microstructures to increase the level of polarization and improve their sensitivity. Yang et al.,^[^ ^253^ ^]^ found that the use of microstructured electrodes strengthens the stress transfer effect, and thus greatly improves the output signal of the pressure sensor.

#### 4.1.4 Triboelectric Effect

In a triboelectric sensor, contact electrification is coupled with electrostatic induction to convert mechanical signals into electrical signals (Figure 3i). Intermittent contact and separation of friction materials with different triboelectric polarities produce electrostatic charges, thereby inducing alternating voltages and currents.^[^ ^247^ ^]^ The quantity of generated electrostatic charges highly depends on the difference in triboelectric polarities of the two friction materials. The polarity of friction materials is defined based on their ability to gain or lose electrons. Several working modes have been proposed for collecting mechanical energy, including vertical contact–separation mode, lateral sliding mode, single‐electrode mode, and freestanding triboelectric‐layer mode.^[^ ^254^ ^]^ Thus, biodegradable triboelectric devices are expected to be used as power supplies for biosensors or to monitor the physiological activities of the human body. Wang et al. proposed an implantable bioresorbable self‐powered pressure sensor based on the triboelectric effect (Figure 3j).^[^ ^63^ ^]^

### 4.2 Principles of Optical Signal Detection

The optical signal, which has high sensitivity and low noise, is advantageous over other physical signals.^[^ ^255^ ^]^ It requires no conductive traces and electrically powered circuit components in the sensing area, which greatly reduces adverse events, and has the potential for compatibility with clinical imaging techniques such as magnetic resonance imaging (MRI).^[^ ^5^ ^]^ Therefore, optical sensors are promising candidates for biomedical applications. Here, we summarized the sensing principles for the detection of optical signals using biodegradable sensors.

#### 4.2.1 Evanescent Field

**Figure 4:**
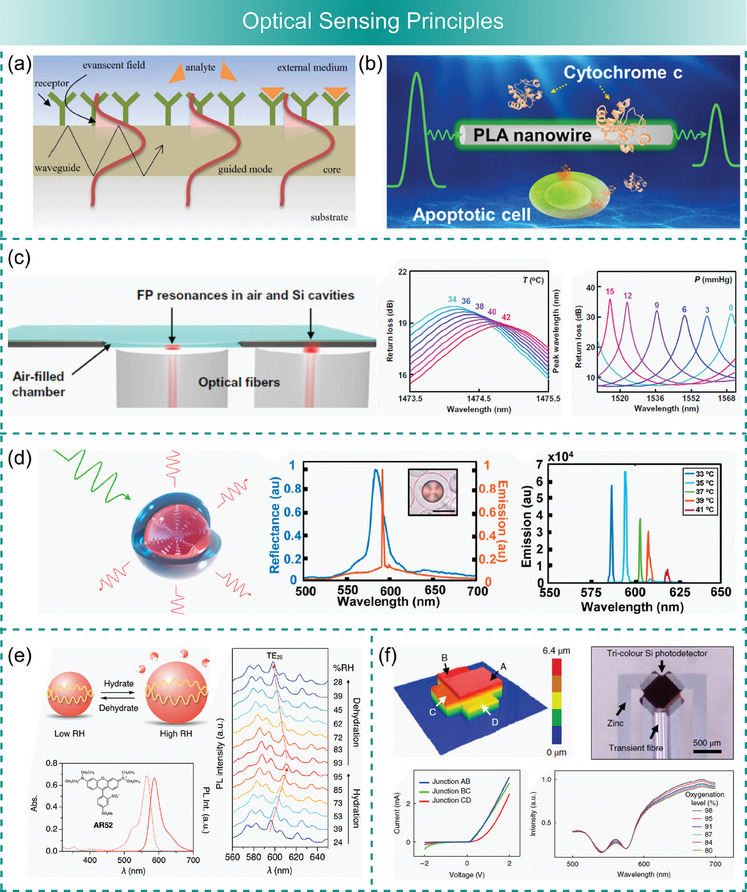
The optical sensing principle of biodegradable physical sensors. a) Schematic of evanescent field sensing. Reproduced with permission.^[^ ^255^ ^]^ Copyright 2020, The Royal Society of Chemistry. b) Schematic of cytochrome c detection with a single PLA nanowire. Reproduced with permission.^[^ ^115^ ^]^ Copyright 2021, American Chemical Society. c) Schematic illustration of a bioresorbable FPI pressure and temperature sensor. The layers of t‐SiO_2_ and Si NM serve as pressure‐sensitive diaphragms that seal an air chamber. The FP resonance peak associated with the air cavity shifts with pressure and temperature changes. Reproduced with permission.^[^ ^5^ ^]^ Copyright 2019 The Authors, some rights reserved; exclusive licensee American Association for the Advancement of Science. d) Bioresorbable microdroplet lasers for biological temperature sensors via complex coacervation. Reproduced with permission.^[^ ^188^ ^]^ Copyright 2021, American Chemical Society. e) Silk fibroin microspheres that expand or contract in response to the surrounding humidity. A peak shift is attributed to moisture absorption by the silk fibroin microsphere, which results in the microsphere diameter increase. Reproduced with permission.^[^ ^38^ ^]^ Copyright 2021, The Royal Society of Chemistry. f) Bioresorbable tri‐color Si photodetector with a bioresorbable fiber optic probe for spectroscopic characterization of biological tissues. Reproduced with permission.^[^ ^2^ ^]^ Copyright 2019, The Author(s), under exclusive license to Springer Nature Limited.

Evanescent wave fiber optic biosensors are a subset of fiber optic biosensors. Light propagates through optical fibers through total internal reflection, where the propagating light is emitted at an angle into the waveguide, and when it reaches the cladding‐core interface, it is reflected and remains in the core. However, it is worth noting that for light reflected at an angle close to the critical angle, a significant portion of the energy extends into the cladding or the medium surrounding the core. The energy spreads in the form of evanescent waves and they extend only up to a short distance from the interface.^[^ ^256^ ^]^ These evanescent waves are sensitive to the changes in the environment surrounding the fiber core. Therefore, optical fibers are often used for highly sensitive sensing and detection of changes in properties or processes occurring at the interface (**Figure** **4**a). Lei et al.,^[^ ^115^ ^]^ demonstrated PLA‐based optical fiber for the detection of cytochrome c with ultrahigh sensitivity by utilizing the modulation of the evanescent wave by the environment. When cytochrome c binds to the PLA nanowire surface, the surface roughness, thickness, and refractive index decrease, resulting in a decrease in light intensity (Figure 4b).

#### 4.2.2 Optical Cavity

Light can be confined into an optical cavity based on interference with itself and form optical resonance. The microcavity can act as an optical signal transducer which mainly employs changes in cavity geometry or material (these may be caused by deforming or heating the cavity, and thus changing the resonance parameters), eventually translating into changes in light intensity or resonant peak shifts.^[^ ^255^ ^]^

A Fabry‐Perot cavity (F‐P) consists of two partially transparent parallel plates with reflective inner surfaces. A cavity is formed with an optical resonance that depends on the distance between the plates. Biodegradable optical pressure sensors can be designed based on this concept.^[^ ^257^ ^]^ Rogers et al.,^[^ ^5^ ^]^ reported bioresorbable Fabry‐Perot interferometers for precise and continuous measurements of pressure and temperature. As shown in Figure 4c, pressure will induce deflections of Si NM which result in changes in the air cavity thickness or the photonic crystal lattice parameters, both of which cause shifts in resonant peak positions within the reflection spectra. These platforms can also be configured to sense temperature by relying on the temperature‐dependent refractive index of silicon. Rogers et al.,^[^ ^105^ ^]^ utilized multiple layers of SiO_x_, SiN_y_, and Si to create a Fabry‐Perot resonance (Si F‐P cavity) with reflection spectrum photonic cavity structures having spectral response dependent on the local temperature. Due to the thermo‐optical effect of constituent materials, temperature changes caused a spectral shift of resonance peaks.

A microsphere cavity exhibits whispering gallery mode (WGM) optical resonance with excellent light confinement efficiency. SF‐based optical microsphere resonators with high sensitivity to humidity variations were developed by Yamamoto et al.,^[^ ^38^ ^]^ Resonant peaks shifted according to the changes in humidity level. Peak shift was attributed to moisture absorption by the SF microsphere, which increases the microsphere diameter (Figure 4e). Rogers et al.,^[^ ^188^ ^]^ designed a microsphere resonant laser cavity based on cholesteric liquid crystal. A temperature‐dependent system was designed based on thermally induced changes in the refractive index (Figure 4d). A conical hollow cavity structure was fabricated by Yuan et al.,^[^ ^258^ ^]^ An interference‐cavity structure was constructed using a section of single‐mode fiber and a section of spider egg sac silk (SESS). As spider silk is a moisture‐sensitive material, variation in ambient humidity will affect the diameter of the SESS, which in turn will affect the length of the interference cavity. A change in the interference cavity length will cause the interference spectrum to redshift and provide the signal for humidity sensing.

Rogers et al.,^[^ ^2^ ^]^ described a photonic device, consisting of three key components: bioresorbable fiber, bioresorbable doped monocrystalline Si‐based photodetector, and zinc electrodes. By comparing light absorption, it can be used for the continuous monitoring of oxygenation and neural activity based on the Beer‐Lambert law. Furthermore, the temperature‐dependent resistance of the Si NM photodetector allows for the monitoring of tissue temperature at a resolution of ≈0.1 °C (Figure 4f).

## 5 Chemical Sensors: Design and Sensing Principles

Chemical sensors made of bioresorbable materials have a huge potential in personalized medicine. Implantable chemical sensors enable real‐time, personalized detection of disease progression and drug efficacy by continuously monitoring the concentration of drugs and target analytes in the body and tissues.^[^ ^32^ ^]^ Typically, the chemical sensor principle involves a reaction between a target analyte and a recognition element, such as enzyme‐substrate binding, antibody‐antigen binding, or acceptor‐donor binding.^[^ ^30^ ^]^ In comparison with physical sensors, chemical sensors require direct contact with the target analytes, and they cannot be completely encapsulated by barrier materials. A major challenge is to measure target chemicals without interference from other chemicals present in biological systems when they are in direct contact with physiological environments without packaging.^[^ ^27^ ^]^ As a result, only a few examples of bioresorbable chemical sensors have been reported. So far, bioresorbable chemical sensors can only detect ions (e.g., pH) or small organic molecules.^[^ ^26^, ^259^ ^]^

### 5.1 Detection of pH

**Figure 5:**
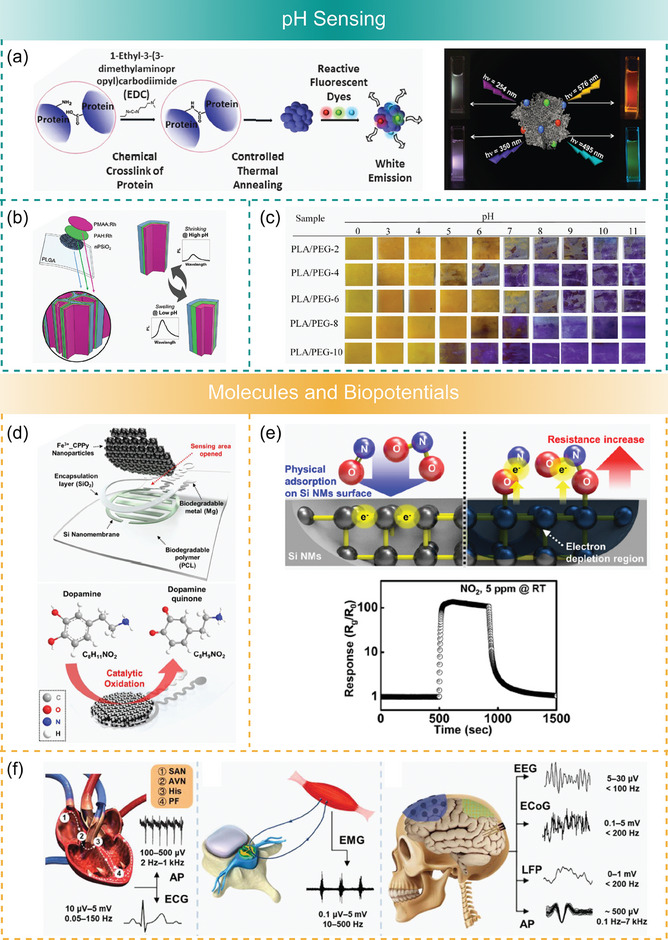
Principles of biodegradable chemical sensors. a) White emitting fluorescent‐dye labeled BSA‐particles for pH sensing. Reproduced with permission.^[^ ^216^ ^]^ Copyright 2016, WILEY‐VCH Verlag GmbH & Co. KGaA, Weinheim. b) Fluorescent pH sensor based on the sensor fluorescence changes with the pH value due to swelling or shrinking of the polymer. Reproduced with permission.^[^ ^32^ ^]^ Copyright 2022, The Authors, Advanced Science published by Wiley‐VCH GmbH. c) Halochromic poly(lactic acid) (PLA) based pH sensor. Reproduced with permission.^[^ ^261^ ^]^ Copyright 2020, Wiley Periodicals LLC. d) Transient, biocompatible hybrid catalyst nanoparticles for implantable dopamine sensor system. Reproduced with permission.^[^ ^259^ ^]^ Copyright 2018, WILEY‐VCH Verlag GmbH & Co. KGaA, Weinheim. e) Bioresorbable SC‐Si NM‐based NO_x_ sensing systems. Reproduced with permission.^[^ ^107^ ^]^ Copyright 2020, The Author(s). f) Recording of different electrophysiological signals with flexible electrodes. Reproduced with permission.^[^ ^263^ ^]^ Copyright 2021, Wiley‐VCH GmbH.

Biodegradable pH sensors are now widely explored. There are several principles explored for pH monitoring, but most of them are based on optical methods. This type of pH sensor can use some specific dyes as indicators and be incorporated into a solid support or matrix. They work by detecting specific changes in the absorption or fluorescence of specific pH indicators based on their protonation or deprotonation at different pH levels. Normally, absorption‐based pH indicators show lower sensitivity than fluorescence‐based pH indicators, but fluorescent pH indicators have limited response ranges and suffer from low solubility in aqueous solutions.^[^ ^32^ ^]^ Kumar et al.,^[^ ^216^ ^]^ reported white‐emitting fluorescent dye‐labeled BSA particles. These sensors emit white light and are composed of three colors in specific intensity ratios, and any increase or decrease in one color intensity can produce detectable, off‐white emissions. The presence of diverse dissociable functional groups (i.e., various pK_a_ values) on the protein particle enhances the sensitivity of the different color intensities to pH and provides a simple way to measure local pH. The sensor is sensitive to pH over a wide range from 2 to 11 (**Figure** **5**a).

pH sensors relying on the swelling or shrinking of certain polymers, which can be detected optically (e.g., by measuring the refractive index) have also been reported.^[^ ^32^ ^]^ Barillaro et al.,^[^ ^32^ ^]^ demonstrated a bioresorbable nanostructured optical pH sensor in which the fluorescence changes linearly with the pH value in the range from 4 to 7.5 due to the swelling or shrinking of the polymer (Figure 5b).

Certain polymers have an intrinsic color that also depends on pH over a rather wide range.^[^ ^260^ ^]^ Marsilla et al.,^[^ ^261^ ^]^ fabricated a halochromic PLA film sensor that is capable of changing color from yellow to purple in response to a pH value in the range from 3 to 11 with a rapid response time of less than a minute (Figure 5c).

Many other principles can be used to sense pH. Borisov et al.,^[^ ^260^ ^]^ summarized these principles in a comprehensive review of optical sensing of pH including methods, materials, and sensor applications. However, the materials used to construct the described optical pH sensors are not fully biodegradable (e.g., photonic crystals which have a structural color that is affected by the local pH value).

### 5.2 Detection of Molecules and Biopotentials

Hwang et al.,^[^ ^259^ ^]^ described a flexible, bioresorbable electrochemical sensor containing Fe NPs coated on Si NMs as a catalyst for monitoring dopamine (DA) (Figure 5d). In another study, the authors used an electronic system based on biodegradable flexible SC‐Si NMs to detect nitric oxide. The principle is based on the changes in the electrical properties of the depletion layer of the n‐type semiconductor sensor through the adsorption and reaction with oxidized gas molecules when SC‐Si NMs react with nitrogen oxides (Figure 5e).^[^ ^107^ ^]^

The human body generates a wide range of electrophysiological signals due to electrical potential changes, including electrocardiogram (ECG), electroencephalogram (EEG), and electromyogram (EMG) signals.^[^ ^262^ ^]^ Electrophysiological signals are the result of electrical activity generated by mass excitable cells on the surfaces of organs. Flexible electrodes with diverse structures and configurations can be used for signal recording. Electrophysiological signal data can be collected through the electrodes and used to gain insight into the health status or to facilitate basic scientific research. Zhang et al.,^[^ ^263^ ^]^ summarized invasive and on‐skin electrodes, and based on the application scenario, both types of electrodes have their advantages and shortcomings, meaning that electrode types should be selected according to the requirements of specific applications (Figure 5f).

Obviously, there are various design and sensing principles that have been successfully utilized in the construction of biodegradable physical and chemical sensors for healthcare monitoring. The appropriate design approaches and sensing principles should be selected and adapted to the specific application. State‐of‐the‐art biodegradable sensors mostly rely on relatively simple and well‐established electro‐mechanical and optical sensing principles coupled with different creative design strategies. Current sensor designs allow for levels of performance comparable to similar non‐degradable sensors. However, due to the limitations of materials selection and available fabrication approaches, some sensing principles remain largely underexplored (e.g., sensing based on magnetic field effects) and more complex electrical or optical sensing configurations still cannot be implemented. Nevertheless, available design and sensing principles have been explored for highly relevant healthcare monitoring applications. We provide an overview of these applications in the next section.

## 6 Applications of Biodegradable Sensors in Healthcare Monitoring

Biodegradable and bioresorbable sensing systems have been developed using the materials, fabrication techniques, and sensing principles mentioned in the previous sections. Up to this point, we can identify that to be able to develop a sensor for a specific application there needs to be thoughtful material selection, device design, and the use of suitable fabrication strategies to achieve the desired performance. The biodegradability and lifetime of such sensing systems have been studied in vitro and in vivo for on‐skin and implantable sensors by monitoring the changes in the vicinity of the sensor location. In this regard, the assessments of biologically relevant parameters such as temperature, pressure, strain, and pH, have been implemented in different settings of the human body such as the integumentary, cardiovascular, nervous, and musculoskeletal systems. This section discusses the applications of physical and chemical biodegradable sensors in detail.

### 6.1 Applications of Physical Sensors

Biodegradable and bioresorbable physical sensors can monitor changes in temperature, pressure, and strain. The continuous assessment of these parameters enables the possibility to follow changes in the human body, either externally (on‐skin applications) or internally (implantable applications). For example, by monitoring pressure changes with wearable devices it is possible to perform real‐time tracking of human motion.^[^ ^264^, ^265^ ^]^ The group of Li developed a flexible piezoresistive skin sensor made of a sandwich structure where an SF/PLGA/polyaniline (SPP) 3D electrospun network is encapsulated on both sides by a K‐carrageenan (KC) solution.^[^ ^127^ ^]^ The network possesses a large surface area and multiple pores that enable the loading of the polyaniline particles providing electrical conductivity for sensing, while the KC improves the mechanical properties of the sensor. The sensor has a sensitivity of 2.54, 1.22, and 0.39 kPa^−1^ in pressure ranges of <41.7, 41.7‐112.2, and 112.2‐165.3 kPa, respectively, with a response time of 160 ms, a limit of detection (LOD) below 100 Pa, and reproducibility over 2000 cycles. The sensor properties and performance allow its implementation in different applications. To monitor the muscle movements during exercise, the sensor was attached to the fingers, wrists, elbows, and knees of the human body. The sensor can detect pressures created by large and small curvature bending produced by the different joints of the human body. The sensor was also installed on the front side of the neck where it was able to sense subtle pressure fluctuations due to vocal cord vibrations caused by the pronunciation of different words. Furthermore, the detection of human cardiovascular function was achieved by monitoring in real‐time the arterial pulse frequency and shape of a sensor placed on the human wrist. The potential to expand the applicability of the sensing system was also demonstrated by attaching the sensor to different parts of a robot to reassemble a human‐computer interface and realizing space resolution pressure detection with a 4×4 sensor array. The degradation of the whole sensor was achieved by immersion in deionized (DI) water at 90 °C. Except for the copper foil used for the external connection of the sensor, all the sensor components decomposed completely after 5 min. The remaining copper foil could be recycled giving this sensor the ability to be environmentally sustainable.

**Figure 6:**
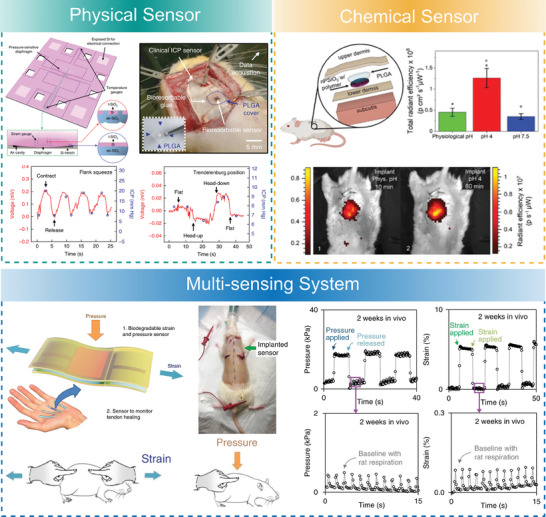
Examples of the implementation of physical, chemical, and multi‐parameter sensing systems for different applications. Physical sensor: piezoresistive silicon sensor for the monitoring of intracranial pressure variation. Reproduced with permission.^[^ ^243^ ^]^ Copyright 2018, The Author(s), under exclusive license to Springer Nature Limited. Chemical sensor: fluorescence‐based sensor for the local detection of pH. Reproduced with permission.^[^ ^32^ ^]^ Copyright 2022, The Authors. Advanced Science published by Wiley‐VCH GmbH. Multi‐sensing system: two stacked capacitive sensors for the independent monitoring of pressure and strain applied for the assessment of forces on muscles. Reproduced with permission.^[^ ^7^ ^]^ Copyright 2018, The Author(s).

Measurements of pressure inside our bodies are relevant for the assessment of the patient's health status and the progression of diseases.^[^ ^266^ ^]^ Of particular interest is the monitoring of intracranial pressure (ICP) after a brain injury.^[^ ^267^ ^]^ The group of Rogers fabricated an inorganic bioresorbable pressure sensor based on silicon nanomembranes.^[^ ^243^ ^]^ The nanomembranes work as strain gauges and form a pressure‐sensitive diaphragm that captures the pressure variations due to the piezoresistive response of the silicon. The diaphragm comprises an electron‐beam evaporated SiO_2_ layer, followed by monocrystalline Si, covered with a thermally grown silicon oxide layer that provides the insulation of the sensing system against biofluids. In vivo monitoring of ICP variations in a rat model was performed for different positions of the rat and during the infusion of mannitol (**Figure** **6**). The sensor was able to monitor the ICP for 18 days with an accuracy of 2 mm Hg and a baseline drift within 1 mm Hg. 25 days after implantation, the signals from the device disappeared due to the dissolution of the bioresorbable metal pads. In vivo bioaccumulation studies combined with hematology and blood chemistry of mice implanted with the bioresorbable intracranial pressure sensor reveal that there were no measurable toxic effects or negative immune reactions. According to the dissolution rates for each component of the sensor, as well as the overall structure of the device, the estimated full dissolution time is ≈400 days. This time could be reduced to ≈290 days without compromising the sensor performance if the thickness of the Si layer is decreased. In addition, it is possible to achieve shorter dissolution times by immersing the sensor in PBS at 95 °C. Complete disintegration of the sensor components is achieved after 80 h in this case. The material constructs and device concept can be implemented for the development of other bioresorbable implants that can be used for sensing motion, flow, and chemical species.

Monitoring of vascular patency after reconstruction surgery opens the possibility of preventing tissue loss.^[^ ^268^ ^]^ By measuring the pressure with a cuff‐type flexible sensor wrapped around arteries, the group of Bao was able to monitor arterial blood flow wirelessly and battery‐free.^[^ ^6^ ^]^ The arteries experience a change in vessel diameter due to the pulsative nature of blood flow. This change in diameter was measured by a capacitive sensor mounted around the artery. The sensor was fabricated by laminating magnesium interconnects over a PGS layer microstructured with pyramids that provide a larger mechanical deformation improving the sensitivity and response time of the sensor. The packing layers of the sensor consist of a bottom layer of PHB/PHV and a top layer of POMaC. In addition, PLA was used as a spacer to prevent electrical short circuits between the elements. The wireless sensing system was designed to be an LCR resonator where the capacitive sensor is connected in series with an inductor coil. The changes in capacitance due to artery expansion produce a shift in the resonance frequency of the LCR circuit, which is measured wirelessly through the skin with an external reader coil. The sensor was tested with an in vivo model where the pulsative behavior of a rat femoral artery was monitored. The pulsation rate calculated from the sensor signal was lower than the one obtained using a Doppler ultrasound system. The discrepancy was attributed to the slower sampling rate of the measuring device (vector network analyzer). After 12 weeks of implantation, all sensor components were degraded except for the PHB/PHV layer. The sensor design and fabrication open the possibility of adaptation for measurements on small and large blood vessels.

Monitoring of temperature inside the body provides information about the infection development, inflammatory status, and other anomalies related to the immune response.^[^ ^269^, ^270^ ^]^ Medical conditions requiring temperature tracking are often temporary. In this regard, the group of Rogers designed a passive sensor able to perform wireless measurements of local temperature changes in targeted internal areas of the body.^[^ ^60^ ^]^ The sensor device consists of an LC‐resonance‐based sensor coupled to an external readout coil. The device is composed of a PEG layer between two Mg foils (parallel plate capacitor), including PLA structures as spacers. The encapsulation is made by PLGA and a wax‐based water barrier. The PEG has a temperature‐dependent dielectric constant that enables the possibility of converting changes in temperature into changes in capacitance. The sensing device was implemented for continuous monitoring of the temperature in the dorsal region and the intracranial space of a rat model. The results showed that while the sensor remained operable for up to 6 days in buffered conditions at 37 °C, for in vivo studies this time was reduced to 4 days. This reduction in operational lifetime could be attributed to the accelerated decomposition of the wax layer resulting from the interaction with enzymes and other species or to the mechanical effects. By comparing the data obtained from the sensor with a standard technique, it was possible to estimate the accuracy of the sensor as 0.5 °C. Interestingly, the presence of the sensors in the dorsal region did not affect the healing, while the head wounds showed imperfect healing due to mechanical stresses applied to the skin. Incubation of the sensor device under buffered conditions in vitro at 37 °C resulted in the dissolution of all components after 69 days except for the wax. Modification of certain sensor parameters, such as the encapsulation layer thickness or the size and design of the coils can provide possibilities to extend the application of this sensing device, not only to different parts of the body but also to the detection of other interesting physical quantities.

### 6.2 Applications of Chemical Sensors

Biodegradable chemical sensors monitor changes in molecule concentration, pH, and gas production. The main challenge for the applicability of biodegradable chemical sensors is stability because the sensing material needs to be in continuous contact with the biofluid of interest. Hence, their typical applications preclude the possibility of full encapsulation. The assessment of the presence of different analytes in body fluids continuously and locally can provide information about the health status and aid in the process of disease diagnosis. For example, changes in the pH value can indicate that our body is suffering alterations.^[^ ^271^, ^272^ ^]^ To assess pH changes locally, the group of Barillaro developed a pH sensor to monitor the pH level through the skin.^[^ ^32^ ^]^ The sensor consists of a nanostructured porous silica membrane layer‐by‐layer coated with two pH‐responsive polyelectrolytes (PAH/PMAA) labeled with Rhodamine‐B. The fluorescence intensity changes with the pH value of the environment surrounding the sensor. This change is caused by the shrinking and swelling of the multilayer stack formed by polyelectrolytes. The calibration curve of the sensor presents a linear behavior in the pH range from 4 to 7.5. The system also exhibits a sensitivity of ‐6.2 ± 1 mV/pH and a resolution below 0.1 pH points over 100 h of continuous operation in vitro. In vivo experiments for real‐time measurement of local pH changes through the skin were performed in the subcutis on the back of mice. Fluorescent images were taken under physiological conditions and at pH 4 (Figure 6). From these images, it was possible to observe an increment in the fluorescence intensity as pH decreased. Full degradation of the sensor was registered one week after implantation, and the biocompatibility studies showed no residual elements from the sensor after two months. The proposed sensing system could be adapted for testing different analytes by coupling the polyelectrolytes with specific bioreceptors.

The presence of specific biomolecules in biofluids, such as neurotransmitters, could be an indicator of disease development.^[^ ^273^, ^274^ ^]^ To monitor dopamine levels, the group of Hwang fabricated a flexible bioresorbable chemical sensor using hybrid NPs of Fe and carboxylated polypyrrole (CPPy) as sensing elements.^[^ ^259^ ^]^ The sensor consists of interdigitated electrodes made of an ultrathin monocrystalline silicon nanomembrane heavily doped with boron coated by hybrid NPs. The electrical contacts were made of Mg, while the encapsulation layer was made of silicon dioxide. When dopamine molecules are present in the testing solution, they are adsorbed on the CPPy surface of the NPs via π‐π interactions. The accumulated dopamine goes through an oxidation process catalyzed by the Fe‐based NPs. The electrons generated during this process transfer to the silicon nanomembrane thereby modulating the electrical characteristics of the sensor. To achieve a spatiotemporal mapping of dopamine secretion in the brain, an array of 5 × 5 sensors was developed. The oxidation of dopamine could produce sensitive variations in response to picomolar concentrations of dopamine. The selectivity of the system was assessed by testing four different neurotransmitters: ascorbic acid, uric acid, epinephrine, and norepinephrine. The interferants produce negligible current changes. Good selectivity of the system is attributed to the formation of π‐π stacking interactions between polypyrrole and dopamine, which is not the case for other neurotransmitters. The degradation of the sensor was evaluated in vitro in PBS at 37 °C and the results showed that sensor components disappeared after 15 h. Even though this study lacks the evaluation of the sensing system in vivo, it demonstrated sensor suitability for operation with biological tissues for continuous real‐time monitoring of biomolecules.

### 6.3 Applications of Multi‐sensing Systems

Healthcare monitoring often requires the assessment of different parameters simultaneously to have a clearer perspective regarding health status. This need is addressed by developing sensing systems that can monitor the changes in two or more parameters. Monitoring of strain and pressure allows to collect information about the forces exerted on different parts of our bodies.^[^ ^275^ ^]^ This is especially relevant for orthopedic applications because it is possible to evaluate the mechanical forces on the muscles after surgery, which will allow for personalized rehabilitation programs.^[^ ^276^ ^]^ An implantable biodegradable sensor for real‐time healing assessment of tendons was reported by the group of Bao.^[^ ^7^ ^]^ The flexible sensing system integrates two vertically stacked sensors that allow independent discrimination of strain and pressure. Monitoring of strain was achieved by measuring the changes in capacitance between two thin‐film Mg comb electrodes that slide relative to each other. Simultaneously, the pressure was measured with a thin, flexible capacitor containing a microstructured elastic PGS dielectric layer. The biodegradable elastomer POMaC was used for packing and PLLA was used as the substrate layer for the Mg electrodes. The sensing system was able to measure strains as low as 0.4% and pressures as low as 12 Pa, with a response time in the millisecond range. Moreover, the response of both sensors could be reproduced thousands of times. Sensor operation was assessed in vitro by immersing the sensor in PBS solution at 25 °C. The sensing response remained stable for up to 3 weeks with a sensitivity comparable to a reference non‐biodegradable sensor. The in vivo performance and biodegradability of the sensor were evaluated by the subcutaneous implantation of the sensor on the backs of rats (Figure 6). It was possible to record signals after 3.5 weeks of implantation since the degradation behavior of POMaC and PGS is based on surface rather than bulk erosion. The animals showed no long‐term inflammatory reaction and good biocompatibility was maintained for 8 weeks after implantation. The sensitivity, stability, and design of the sensor illustrate the concept of a sensing system not only relevant for orthopedic applications but also for other applications where monitoring of mechanical deformations and pressures in real‐time is relevant.

Localized monitoring of tissues and biofluids for the assessment of health status and metabolic activity in different regions of our bodies can assist during and after surgical intervention procedures. The group of Rogers developed injectable photonic devices for spectroscopic determination of cerebral temperature, oxygenation, and neural activity.^[^ ^2^ ^]^ The sensor consists of a PLGA substrate with a Si nanomembrane photodetector, Zn electrodes, a bioresorbable PLGA fiber, and a silicon oxide encapsulation layer. The incorporation of a tunable laser for the Si nanomembrane produces transmission spectra of blood samples at different oxygenation levels, showing a linear relationship between the photocurrent and oxygen saturation. The Si nanomembrane photodetector possesses a temperature‐dependent resistance that allows monitoring of the tissue temperature with a resolution of 0.1 °C. Additionally, a bioresorbable optical filter made of alternating layers of SiO_x_ and SiN_x_ was integrated on top of the photodetector to achieve the detection of fluorescence‐based calcium indicators of neural activity. The performance of the sensor was evaluated in live animal models for continuous measurement of temperature, oxygen saturation, and Ca^2+^ concentration, while the animals moved in cage environments. The sensor was able to record temperature changes related to metabolic activity, changes in blood oxygenation in response to variations of the environmental oxygen concentration, and a reduction in calcium levels when using anesthesia with 1.7% isoflurane. Studies of biodistribution, blood chemistry, and hematology showed no abnormal accumulation of the materials in the organs, as well as no tissue damage related to the implantation of the sensor, no measurable toxic effects, and absence of negative immune response. Finally, the encapsulation layer of SiO_2_ enabled the stable operation of the sensor for 10 days. The photonic implantable technology reported in this work provides interesting approaches for the realization of sensing platforms that can have relevance in disease pathology research.

In healthcare monitoring, the successful implementation of biodegradable sensing systems relies on the careful selection of materials, fabrication techniques, and sensing principles to ensure optimal performance. Evaluating the feasibility of these sensors requires conducting both in vitro and in vivo tests. Currently, most sensing systems rely on physical connections for operation. However, the ultimate objective is to develop biodegradable sensors that can function autonomously without the need for tethering, enabling continuous monitoring in on‐skin or implantable applications. In the following section, we review some of the available biodegradable powering systems that can drive the development of fully biodegradable sensing platforms.

## 7 Power Sources

A major challenge in biodegradable sensors is the realization of adequate biodegradable powering systems necessary for sustained device operation during its intended lifetime. In the case of supplying electrical power, one approach is the use of energy storage devices such as batteries and supercapacitors, while the alternatives comprise energy harvesters or systems for wireless power transfer.

### 7.1 Biodegradable Batteries

Batteries are commonly used as stable and low‐noise temporary power sources in portable electronic systems. Their interfacing with electronic systems is simple and well‐established. With the growing use of portable devices in different application areas, disposal of used non‐degradable batteries has become a serious environmental issue and the replacement of batteries in implanted medical devices requires additional surgical interventions carrying risks for the patient. Therefore, the demand for reliable, biodegradable, and green batteries will continue to rise inside and outside of the healthcare sector.^[^ ^277^ ^]^ Degradable batteries in wearable and implantable sensors should match the mechanical properties of the surrounding tissues, have as small a size as possible, and be able to deliver sufficient power during the life cycle of the sensor.

**Figure 7:**
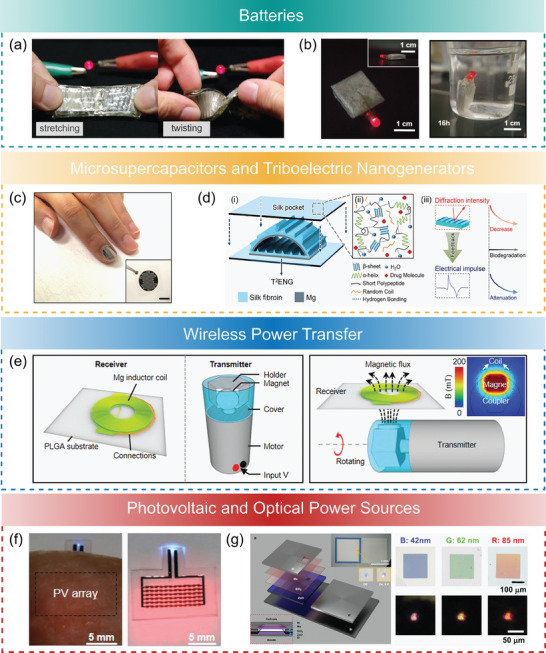
Examples of different power sources used in biodegradable sensing devices. a) Flexible and stretchable bioresorbable battery for wearable sensors. Reproduced with permission.^[^ ^65^ ^]^ Copyright 2022, The Authors. Advanced Materials published by Wiley‐VCH GmbH. b) Biodegradable battery for implantable sensors. Reproduced with permission.^[^ ^19^ ^]^ Copyright 2018, WILEY‐VCH Verlag GmbH & Co. KGaA, Weinheim. c) Microsupercapacitor for in vivo powering applications. Reproduced with permission.^[^ ^80^ ^]^ Copyright 2017, WILEY‐VCH Verlag GmbH & Co. KGaA, Weinheim. d) Transient triboelectric nanogenerators for self‐powered in vivo monitoring devices. Reproduced with permission.^[^ ^182^ ^]^ Copyright 2018, WILEY‐VCH Verlag GmbH & Co. KGaA, Weinheim. e) Wireless power transfer based on a rotating magnet as a transmitter and bioresorbable antenna as a receiver. Reproduced with permission.^[^ ^23^ ^]^ Copyright 2019, WILEY‐VCH Verlag GmbH & Co. KGaA, Weinheim. f) Implantable and bioresorbable photovoltaic array as a power supply relying on an external optical power source. Reproduced with permission.^[^ ^20^ ^]^ Copyright 2018, WILEY‐VCH Verlag GmbH & Co. KGaA, Weinheim. g) Fully bioresorbable transient light‐emitting diode for the powering of implantable optical sensors. Reproduced with permission.^[^ ^21^ ^]^ Copyright 2019, WILEY‐VCH Verlag GmbH & Co. KGaA, Weinheim.

Huang et al.,^[^ ^19^ ^]^ reported a fully bioresorbable battery (**Figure** **7**b) based on the Mg anode, MoO_3_ cathode, and an alginate hydrogel electrolyte. The battery comprised stacked layers of Mg foil (anode, 50 or 200 µm), alginate hydrogel prepared in phosphate‐buffered solution and cross‐linked with CaCl_2_ (≈ mm), MoO3 paste formed by mixing PLGA binder and MoO_3_ powder (cathode, 50 µm), and Mo foil (current collector, 5 or 30 µm) with sputtered MoO_3_ thin film (1 µm). The battery was further encapsulated by thin layers of PLGA and polyanhydride. The battery could achieve a voltage output of 1.6 V for up to 48 h for the thickest PLGA/MoO_3_ paste layer (350 µm) at a discharge current density of 0.025 mA cm^−2^. With a 200 µm thick layer of MoO_3_ paste, voltage above 1.45 V could be sustained for more than 50 h (until MoO_3_ was depleted) followed by a drop to the value of 0.6 V (Mo foil acting as cathode) which could be retained for additional 250 h, indicating the energy capacity of 6.5 mWh cm^−2^. The high voltage in the initial period could be retained up to the discharge current density of 0.15 mA cm^−2^. With the encapsulation layers, the operational lifetime of the battery could reach 13 days. It was demonstrated that the battery can power a standard calculator or low‐power ECG amplifier, and light up a red LED for up to 16 h in PBS. The battery showed remarkable and safe degradation performance in vitro and in vivo. 50 µm thick Mg and 5 µm thick Mo foils were used for all degradation experiments. Most battery components could degrade in vitro after 9 days in PBS under physiological conditions, but Mo required 10 more days of accelerated degradation at 85 °C to completely dissolve. PLGA/MoO_3_ paste encapsulated with PLGA was co‐incubated with L‐929 mouse fibroblast cells to examine the biocompatibility of the cathode material. The results indicated excellent biocompatibility of cathode material and even the improved growth ability of L‐929 cells. In vivo subcutaneous implantation of batteries in a rat model resulted in full implant dissolution after 4 weeks without any detectable reaction of the tissue to the implant or its degradation products.

Karami‐Mosammam et al.,^[^ ^65^ ^]^ fabricated the first stretchable and biodegradable battery (Figure 7a). The authors combined laser‐assisted kirigami patterning of Mg (anode, 50 µm) and Mo foils (current collector, 50 µm) with stretchable calcium alginate hydrogel separator (1 mm), xanthan gum/MoO_3_ paste (cathode), and stretchable PGS gel enclosure (1 mm) as a layer stack to form a fully degradable, stretchable, and functional battery. The stretching ability of the battery was limited by the separator. Under 20% uniaxial strain, the battery could survive more than 900 stretching cycles. The battery exhibited an energy density of up to 1.72 mWh cm^−2^ (under 45 µA cm^−2^ of discharge current) which is comparable to the biodegradable non‐stretchable counterparts. Under ambient conditions, mechanically and electrically unloaded batteries could sustain a voltage above 1.5 V for 77 h. Even under 20% of constant strain at a discharge current of 200 µA, the voltage level stayed above 1 V for 2.4 h. 20% of cyclic straining caused slight voltage oscillations dictated by changes in internal resistance. The battery could supply 200 µA of current to the load even after repetitive biaxial deformation for 30 h. The performance of the battery under deformation was demonstrated by powering a digital thermometer and its potential utility was shown by powering a wearable patch for sodium sensing in sweat for several hours with an average consumption current of 5 µA. The degradation of the battery was demonstrated in vitro in PBS and simulated gastric acid under physiological conditions. Due to the very different degradation properties of battery components, the assembled battery degrades by more than 70% (as measured by mass loss) after 95 days in simulated gastric acid and 73 days in PBS.

Another notable approach to constructing stretchable batteries for wearable applications taken by Ma et al.,^[^ ^196^ ^]^ is the joining of composite starch‐based hydrogel layers to create a galvanic cell (containing added Zn powder in one layer and added combination of Cu and CuCl_2_ powders in the other layer) with a voltage of 0.81 V and strain‐sensitive behavior. The hydrogels with high amylose content comprising glycerol and CaCl_2_ exhibited remarkable flexibility, stretchability, and self‐healing, while also retaining the ability for simple reprocessing. Their high sensitivity to low compression stress levels (1.5371 kPa^−1^) enabled effective sensing of subtle strain changes present in wrist pulsation and throat vibration. The starch‐based hydrogels efficiently degraded more than 85% in nutritional soil after 40 days.

### 7.2 Alternative Biodegradable Sources of Electrical Power

Lee et al.,^[^ ^80^ ^]^ demonstrated fully biodegradable MSCs (Figure 7c) with significantly enhanced electrochemical performance due to the pseudocapacitive behavior of continuously growing metal‐oxide layer as a result of electrochemical corrosion during charge/discharge cycles. They fabricated MSCs comprising electrodes and current collectors made of degradable metals (Mo, W, or Fe), agarose mixed with NaCl as a gel electrolyte, and PLGA as the degradable substrate. The total thickness of the device was ≈ 160 µm. When using interdigitated Mo electrodes, the MSCs reached an areal capacitance of 1.6 mF cm^−2^, energy density of 0.14 µWh cm^−2^, and power density of 1 mW cm^−2^ (properties comparable to non‐degradable MSCs). The stack of 3 MSCs charged at 3.3 V in 7 s could light up a red LED for several seconds. The device could maintain stable operation in PBS under physiological conditions for 6 h when encapsulated with 10 µm of PLGA or even for days when encapsulated with polyanhydride. The degradation rate in vitro was limited by the PLGA substrate (weeks to months) and the agarose gel (requires enzymes or non‐physiological conditions such as pH = 12).

Zhang et al.,^[^ ^182^ ^]^ reported transient triboelectric nanogenerators based on a special configuration of silk and Mg layers (Figure 7d). By tuning the silk layers in terms of molecular weight, surface structuring, and encapsulation matrix properties, it was possible to customize the operating lifetime of the power supply and the triboelectric output voltages, while also monitoring the structural integrity of the device. Under optimal conditions, the device was able to achieve an open circuit voltage of 60 V, short‐circuit current of 1 µA, and a power density of 38.5 mW/m^2^ under 100 MΩ load. Under 90% humidity, device operation could be sustained for up to 10 h, while Mg layers typically degraded after 6 h in DI water. Depending on the silk layer properties, partial or complete resorption was observed several weeks after in vivo implantation in mice.

Guo et al.,^[^ ^23^ ^]^ introduced a fully bioresorbable system for wireless power transfer at frequencies below 200 Hz (Figure 7e). The system operates as a magnetic energy harvester comprising a rotating permanent magnet electromagnetically coupled to a completely degradable receiver suitable for implantation or external use (e.g., on the skin). The energy transmitter part of the system contained a neodymium disc magnet (diameter: 9 mm; thickness: 5 mm) rotated by an electric motor equipped with a speed controller. The biodegradable receiver comprised a stack of two planar 30 µm thick Mg coils separated by the PLGA dielectric layer. Rotation of the permanent magnet induced the voltage in Mg coils according to Faraday's law in a frequency‐dependent manner. Compared to other wireless powering options, the major advantages of this approach are the elimination of impedance matching requirements and avoidance of electromagnetic radio‐frequency exposure. At a distance between the transmitter and receiver of 4 mm and the harvester working area of 0.78 cm^2^, the peak output power density at 51 Hz was 8.7 mW cm^−2^. The addition of a magnetic field concentrator (MFC) (composite of PLGA and 50‐nm‐sized Fe_2_O_3_ nanoparticles) improved the induced voltage by ≈ 14% and enhanced the reliability at misalignments of up to 6 mm. The vertical stack of 5 Mg coils in a receiver enabled the powering of a single LED (open‐circuit peak‐to‐peak voltage of 3.26 V). The receiver with MFC encapsulated in PLGA could retain its functionality in DI water for only up to 10 h, while it degraded within several weeks in PBS under elevated temperature (pH 7, 50 °C).

Externally applied optical power can also be exploited to create a temporary photovoltaic power supply. Lu et al.,^[^ ^20^ ^]^ demonstrated a fully resorbable power supply in the form of an array of thin monocrystalline Si‐based photovoltaic microcells (Figure 7f). The array contained 72 microcells interconnected with a Mo metallization pattern (1.5 µm), each consisting of interpenetrating boron and phosphorus‐doped Si regions (1.5 µm) encapsulated by buried SiO_2_ (3 µm, bottom) and drop‐casted PLGA (200 µm, top). Output voltage and current of the power supply could be tuned by modifying the network geometry of Mo interconnects. To test the realistic performance of the power supply, the output power characteristic was tested under different illuminations without any cover and when covered by porcine skin and fat tissue (both ≈2 mm thick). The power output was reduced from 122 µW (no cover) to 25 µW (porcine skin and fat layers) under 100 mW cm^−2^ of 1 sun illumination meaning that only ≈ 19% penetrates the photovoltaic microcell array. When NIR illumination was used at an optical power density of 200 mW cm^−2^ and wavelength of 780 nm, the output power was reduced from 242 µW to 64.4 µW (sufficient to operate an LED or a pacemaker). The power supply could light up a blue LED for 5 days in vitro under physiological conditions in PBS or 3 days in vivo when implanted in the infrascapular region of adult rats. Biocompatibility of the system was demonstrated in vitro by culturing human umbilical vein endothelial cells. The system was also fully resorbed in vivo 4 months after subcutaneous implantation in adult rats with no registered inflammation of surrounding tissues.

### 7.3 Biodegradable Sources of Optical Power

The delivery of optical power is critical for many optical sensing approaches and thus biodegradable light sources are necessary to complete a fully degradable system. The use of biodegradable optical sensors often implies the use of dedicated optical power (light) sources required to perform the measurement and produce sufficiently strong signals for accurate and reliable readout. Except for some optical sensing approaches relying on luminescent or halochromic properties of biodegradable sensing elements, most biodegradable sensors rely on external light sources. Because of the immense challenges related to the development of fully degradable light sources, only a single major contribution has been reported in the literature.

Lu et al.,^[^ ^21^ ^]^ demonstrated a transient light‐emitting diode (LED) that safely degrades in aqueous solutions (Figure 7g). This LED was constructed by forming a 200 nm thick ZnO layer using pulsed laser deposition on a thin Si substrate (12 µm). By applying conventional microfabrication techniques, the active area of the ZnO layer (20 µm × 20 µm) was electrically contacted using thin layers of Mo (8 nm, transparent layer) and W (100 nm). A thin layer of patterned SiO_2_ (15 nm) was applied as an insulation layer for the LED. Biodegradable conductive paste and Mg wires were used to interface with the external power supply. The fabricated LED exhibited a threshold voltage of 5 V and emitted white light in the broad range of visible wavelengths at the highest optical power density of 0.7 mW cm^−2^ under 9 V excitation. Due to the broad emission band of ZnO, different colors can be selected using degradable Fabry‐Perot optical filters in the form of freestanding monocrystalline silicon nanomembranes. The color of the emitted light can be adjusted by selecting the appropriate membrane thickness (red, 85 nm; green, 62 nm; and blue, 42 nm). The authors investigated the in vitro degradation of the LED in PBS at 37 °C. Although almost uniform degradation of different layers was observed, full device dissolution was estimated based on Arrhenius scaling to occur only after several years.

## 8 Conclusions

The last decade has witnessed significant advances in biodegradable sensing technologies, which led to the development of fully degradable electrical and optical basic sensing systems in healthcare with comparable functionality to their non‐degradable counterparts. These systems emerged to fill the application gap requiring temporary monitoring of health parameters in a manner that reduces risks for the patients and addresses recent environmental concerns. Many of the developed biodegradable sensing systems demonstrate clear utility in vitro and some of them in vivo in animal models. This is evidenced by the technology readiness levels (TRL)^[^ ^278^ ^]^ reaching from 2 (technology concept and/or application formulated) to 4 (component and/or system validation in a laboratory environment). There are several key challenges left to overcome before reaching the level of complete functional devices for healthcare monitoring, such as adequate performance of in vivo power sources, sensor component integration, controlled on‐demand transiency, and effective (bio)chemical sensing. Further advances in the areas of biomaterials research, intelligent sensor design, and cost‐effective fabrication techniques compatible with degradable materials are required to construct complete sensing systems of higher complexity and scale up their use to the commercial level. Biodegradable and bioresorbable healthcare sensors present one of the key frontiers in science and engineering with promising potential to simultaneously meet important clinical needs and demands of environmental safety. These sensing devices will have a tremendous impact in all healthcare scenarios where temporary real‐time and continuous monitoring is required to ensure good quality of patient care, and especially in applications where reduction of surgical risk is critical or unfavorable for patient outcomes. It is expected that the field of biodegradable sensors will experience considerable growth in the coming years and become one of the focal points of future research efforts in healthcare. A multidisciplinary and synergistic approach is necessary to ensure the continuous growth of this field and enable the matching of its development with market needs.

## 9 Challenges and Future Perspectives of Biodegradable Sensors in Healthcare

Thanks to the remarkable recent progress in the development of biodegradable sensors, certain basic sensing systems have demonstrated successful operation, biocompatibility, and in vivo biodegradability in animal models, and thus can be regarded as proofs‐of‐concept for healthcare monitoring applications. The reviewed contributions can be placed in the range of TRLs between 2 and 4. While there is still no evidence of clinical translation for biodegradable sensors, successful examples of in vivo applications provide a good insight into their sensing behavior and great potential.

Although significant progress has been made in the field of biodegradable healthcare sensors in recent years, there are still significant barriers to overcome in the development of fully functional transient sensing devices for healthcare monitoring. In this context, we emphasize the critical challenges spanning different levels of sensor design and development, while also considering possible perspectives and directions for further research.

### 9.1 Material Level

In most of the developed biodegradable sensors, degradation occurs spontaneously following a passive mechanism mainly determined by the encapsulation layer properties and inherently slowly degrading materials constituting certain sensor components. Typically, there is a significant mismatch between the desired operational lifetime of the sensor and its life cycle completed after full degradation. The issue of long degradation times compared to useful operation times is particularly emphasized in power sources and becomes even more problematic for bulky components (e.g., batteries). In addition, degradation can only be roughly predicted based on the accumulated knowledge about material properties. Achievement of desired degradation profile can thus be difficult, due to inherent variability between patients and the evolution of monitored healthcare parameters. To overcome these issues, viable approaches for triggered on‐demand degradation of responsive materials within the sensor should be introduced and they already are a topic of active research.^[^ ^24^, ^25^, ^279^ ^]^

In terms of novel material development, there is a need to bridge the design and manufacturing gap in the field of biodegradable sensors by engineering suitable soft and flexible conducting and semiconducting materials. Progress in such materials is expected to come from advanced molecular design. As an alternative, the engineering of polymer‐based composites with soft functional fillers^[^ ^280^, ^281^ ^]^ can be further explored. In the case of implantable biodegradable sensors, attention should also be directed toward developing materials and tagging techniques compatible with medical imaging.

Another important and insufficiently explored potential issue is the cumulative toxicity of biodegradable materials and their products at the local and systemic level in humans and the environment. The potential for associated acute and chronic harmful effects needs to be thoroughly studied in the future.

### 9.2 Fabrication Level

The nature of biodegradable materials limits the range and performance of fabrication techniques that can be applied in the manufacturing of biodegradable sensing devices. These materials are commonly chemically reactive, susceptible to dissolution in aqueous environments, and sensitive to processing at high temperatures or in a vacuum. In addition, many biodegradable materials are shaped as soft and flexible layers making the high‐resolution patterning of complex and small structures more difficult. To reach higher levels of integration using versatile materials typically constituting biodegradable sensors, it is necessary to develop innovative, rapid, and low‐cost printing techniques compatible with important classes of soft biodegradable materials. Such techniques could be used in synergy with conventional microfabrication techniques for electronics (applicable to e.g., Si‐based materials) to build hybrid high‐performance sensing devices.

### 9.3 Sensor Level

While a great variety of biodegradable physical sensors based on electrical and optical principles has been demonstrated, chemical sensors are still limited to sensing of pH, ions, and small molecules. For adequate diagnostic performance, it is crucial to enable the monitoring of biomolecules and metabolites as important biomarkers in healthcare. To achieve this goal, it is necessary to develop innovative sensing materials or functionalization methods, which produce specific interactions with target analytes and reach reliable temporary operation in contact with relevant biofluids. An inherent property of biodegradable sensors, the degradation of constituting materials, can be envisioned as a useful measure in novel alternative chemical sensing approaches. The implementation of eco‐sustainable and biodegradable devices exploiting magnetic field‐based sensors also remains underexplored. Although Fe is a ferromagnetic, biocompatible, and biodegradable material, its oxidation presents a challenge for reliable sensor performance. We envision that this problem can be solved by applying printing technologies that enable the incorporation of Fe nano‐ and micro‐particles within biocompatible and biodegradable matrices while using suitable chemical approaches to prevent Fe oxidation and establish electrical percolation in the printed composite. The use of self‐healing approaches based on alternating magnetic fields^[^ ^282^ ^]^ also seems to be advantageous for improving the electrical performance of such devices. Magnetic‐field‐based sensors could be a particularly useful component of wireless implantable healthcare monitoring devices.^[^ ^158^ ^]^

An examination of the principles used in currently implemented biodegradable sensors for healthcare monitoring reveals that developed optical sensors fall behind compared to their electrical counterparts. This is presumably a consequence of generally more demanding fabrication constraints, disadvantages associated with significant light absorption in human tissues, and the difficulty of constructing sufficiently effective light sources for in vivo applications. Further development of complete functional optical sensing devices remains an open and important challenge to be addressed by future research.

### 9.4 Application Level

Application‐specific issues in the realization of biodegradable sensors are mainly related to power sources and data transfer. Many developed sensors still must rely on physical connections via cables or fibers to ensure sufficient power for operation and data acquisition. In the ideal scenario, biodegradable sensing devices should be stand‐alone and tether‐free.

To date, developed bioresorbable batteries do not provide sufficient energy density and an operational lifetime to continuously power sensing devices (especially when implanted). Alternative electrical power sources (MSCs, energy harvesters, and photovoltaic cells) cannot sustain the delivery of sufficient energy amounts and are more suitable as backup power supplies which can be envisioned as a solution in the future if coupled with a rechargeable battery or similar energy storage device.

An alternative for powering and data transmission is the use of wireless energy or data transfer. Such wireless systems are useful for external on‐skin sensors and subcutaneously implanted sensing devices. However, wireless signals suffer from poor propagation through biological tissues and their use remains limited to transmitter‐receiver distances of up to a few cm. Therefore, powering and data collection from deeper tissue structures such as sensors placed on blood vessels or within the brain remain challenging. Extension of the propagation range for wireless signals with innovative coupling techniques is an attractive area of research related to biodegradable sensing systems in healthcare. Probably one of the most prominent challenges for the full realization of biodegradable sensing devices is the realization of degradable analog integrated electronic circuits enabling signal amplification, filtering, processing, and frequency modulation near the detection site (particularly in vivo). Realization of these electronic modules must start from the main building blocks including reliable passive components (resistors and capacitors) and key active components of higher complexity (operational amplifiers) to achieve significant advances. It is crucial to acknowledge that the effects of continuous energy dissipation (e.g., heat and radiation) during the operation lifetime of biodegradable sensors on the surrounding tissues were not yet clearly quantified. Studies related to the long‐term in vivo safety assessment of biodegradable sensors are thus required in the future to verify design and development strategies.

Finally, it is important to note the perspective of knowledge transfer pertinent to biodegradable sensor development between different research communities. Although this review focuses on human healthcare monitoring, biodegradable sensing devices are also being actively developed for monitoring applications in precision agriculture and ecology.^[^ ^283^, ^284^, ^285^ ^]^ In addition, it may be of advantage to combine biodegradable sensors with tailored biodegradable soft actuators^[^ ^286^ ^]^ in future devices to enable advanced therapeutic applications or precise triggering of degradation processes. The synergy of concepts and approaches from these different research fields can further accelerate the progress of biodegradable sensor development and broaden the scope of potential applications.

## Conflict of Interest

The authors declare no conflict of interest.
